# A Regional-Scale Ocean Health Index for Brazil

**DOI:** 10.1371/journal.pone.0092589

**Published:** 2014-04-02

**Authors:** Cristiane T. Elfes, Catherine Longo, Benjamin S. Halpern, Darren Hardy, Courtney Scarborough, Benjamin D. Best, Tiago Pinheiro, Guilherme F. Dutra

**Affiliations:** 1 Department of Ecology, Evolution and Marine Biology, University of California Santa Barbara, Santa Barbara, California, United States of America; 2 National Center for Ecological Analysis and Synthesis, Santa Barbara, California, United States of America; 3 Bren School of Environmental Science and Management, University of California Santa Barbara, Santa Barbara, California, United States of America; 4 Imperial College London, Silwood Park Campus, Berkshire, London, United Kingdom; 5 Digital Library Systems, Stanford University, Stanford, California, United States of America; 6 Atlantic Forest Program, Conservation International Brazil, Belo Horizonte, Minas Gerais, Brazil; 7 Marine Program, Conservation International Brazil, Rio de Janeiro, Rio de Janeiro, Brazil; Aristotle University of Thessaloniki, Greece

## Abstract

Brazil has one of the largest and fastest growing economies and one of the largest coastlines in the world, making human use and enjoyment of coastal and marine resources of fundamental importance to the country. Integrated assessments of ocean health are needed to understand the condition of a range of benefits that humans derive from marine systems and to evaluate where attention should be focused to improve the health of these systems. Here we describe the first such assessment for Brazil at both national and state levels. We applied the Ocean Health Index framework, which evaluates ten public goals for healthy oceans. Despite refinements of input data and model formulations, the national score of 60 (out of 100) was highly congruent with the previous global assessment for Brazil of 62. Variability in scores among coastal states was most striking for goals related to mariculture, protected areas, tourism, and clean waters. Extractive goals, including Food Provision, received low scores relative to habitat-related goals, such as Biodiversity. This study demonstrates the applicability of the Ocean Health Index at a regional scale, and its usefulness in highlighting existing data and knowledge gaps and identifying key policy and management recommendations. To improve Brazil's ocean health, this study suggests that future actions should focus on: enhancing fisheries management, expanding marine protected areas, and monitoring coastal habitats.

## Introduction

Brazil's coastline spans more than 7,000 km with a vast diversity of ecosystems, including extensive mangrove areas in the Amazon basin, coral reefs in the Northeast, and lagoons, estuaries and saltmarshes in the south. These systems play a fundamental role in the economy and identity of the country. As Brazil's economy continues to grow – for 2012 it was listed as the seventh largest economy in the world [1] – interest in using and benefiting from coastal and marine systems is also growing. Often the various activities tied to these benefits, such as fisheries, coastal development and tourism, come into conflict, and resource managers and policy makers are faced with decisions about how and where to allow and regulate each of them, with the ultimate goal of maintaining and ideally improving the overall health of the ocean and the communities that use it.

Given this context, there is a great need in Brazil for tools to assess and monitor the overall health of coastal ecosystems, as well as the status of components of the system. A framework was recently developed to do just that, and was applied to every coastal country in the world [2]. This index to assess the health and benefits of the ocean (Ocean Health Index) evaluates the condition of coupled human-ocean systems by tracking the current status and likely future state of ten publicly held goals, ranging from food provision to jobs, tourism, and coastal protection (Table 1).

**Table 1:** Ten public goals and sub-goals showing benefit measured under each.

Goal	Subgoal	Benefit measured
Food Provision (FP)	Fisheries (FIS)	Seafood sustainably harvested for human consumption from wild, or cultured stocks
	Mariculture (MAR)	
Artisanal fishing opportunity (AO)		Opportunity to engage in artisanal fishing as a social, cultural and livelihood activity
Natural products (NP)		Amount of sustainably harvested natural products (other than for food provision)
Carbon storage (CS)		Conservation of coastal habitats affording carbon storage and sequestration
Coastal protection (CP)		Conservation of coastal habitats affording protection from inundation and erosion
Tourism and recreation (TR)		Opportunity to enjoy coastal areas for recreation for locals and tourists
Coastal livelihoods and economies (LE)	Livelihoods (LIV)	Employment (livelihoods) and revenues (economies) from marine-related sectors
	Economies (ECO)	
Sense of place (SP)	Iconic species (ICO)	Sense of place and cultural connectedness to the ocean afforded by lasting special places and iconic species
	Lasting special places (LSP)	
Clean waters (CW)		Clean waters that are free from pollution, debris and safe to swim in
Biodiversity (BD)	Habitats (HAB)	Conservation of biodiversity of species and habitats for their existence value
	Species (SPP)	

The Index is based on the understanding that humans are part of ecosystems and that the health of natural and human systems are tightly coupled [3], [4]. From this coupled human-natural systems perspective, a healthy ocean is defined as one that provides a range of benefits to people now and in the future [2]. As such, the Index measures the amount of benefits relative to a sustainable optimum. The Index is not intended to be a measure of how pristine an area of ocean or coastline is.

The novelty of the Index is that it provides an integrated framework in which to quantitatively assess and compare the condition of these benefits, thus providing a portfolio perspective useful for informing management decisions. The Index can also be used to track progress in achieving specific management goals, because it establishes a target or reference point to which current status and likely future condition are compared ([2], [5], Table S1 in Text S1).

Here we present a case study, applying the Ocean Health Index framework to Brazil at the national and sub-national levels. The global analysis [2] precluded use of higher resolution datasets that are available for individual countries, data that can provide a more accurate assessment of a country's ocean health as well as sub-national assessments. As such, the global analysis is too coarse to guide specific interventions at national and regional levels, particularly for a country as large and heterogeneous as Brazil.

Applying the Ocean Health Index to Brazil provides an important opportunity to test the scalability and flexibility of the Index to be adapted to country-specific concerns by including higher resolution information, place-specific targets and regional proxies for calculating goals. The case study also highlights a number of challenges related to data quality and quantity for assessing the range of benefits evaluated under the Index framework. Here, we show how the Index can be adapted to the Brazilian context, and discuss the main patterns and policy implications emerging from our analysis. Our intent is that the lessons learned from this case study can be used to guide future assessments and management strategies in Brazil, and help to inform other current and future regional applications of the Ocean Health Index.

## Methods

Details on calculation of the Index are provided in Halpern *et al.* [2]. Here we give a brief summary, and elaborate on goal-specific methods and data layers used in this case study in Text S1.

The Index is comprised of ten widely-held public goals: Food Provision, Artisanal Opportunities, Natural Products, Carbon Storage, Coastal Protection, Coastal Livelihoods and Economies, Tourism and Recreation, Sense of Place, Clean Waters and Biodiversity (Table 1). As the Index is focused on the sustainable provision of benefits, we do not include activities such as oil and gas exploration. The location of oil and gas deposits and productivity of such reserves are not indications of a healthy, or sustainably managed ocean. It is worth noting, however, that oil is incorporated as a pollutant in the pressure calculations (see below) and the status of the Clean Waters goal.

For each goal, a score is calculated from four dimensions – current status, recent trend, existing pressures and expected resilience in the near-term based on current management actions. The Index value (*I*) is determined as a linear weighted sum of the scores for each of the public goal indices (*I_1_*, *I_2_*,…, *I_10_*) and the appropriate weights for each of the goals (α_1_, α_2_,…α_10_), such that: 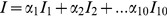
(1)


The weights determine the relative importance of each goal in the overall Index score and ideally reflect people's values within the region. Here we used equal weighting, as an in-depth interview process with stakeholders from all Brazilian coastal states was outside the scope of this case study (for an example, see [6]).

Each goal score, *I_i_*, is calculated as the average of its present status *x_i_*, and an estimate of its likely near-term future status 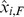, such that:
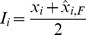
(2)


The present status of goal *i*, *x_i_*, is its present status value, (*X_i_*), relative to a reference point, *X_i, R_* uniquely chosen for each goal and scaled 0 to 100. 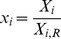
(3)


The reference point, X_i, R_, is determined a number of ways depending on the purpose (management objective) and data constraints of each goal. The main ways of establishing a reference point are: through a known functional relationship (e.g. a target value of extracting the maximum sustainable yield of a given fish stock), a time series approach (e.g. historical habitat extent), a spatial comparison (e.g. the country with highest wages in marine-related sectors), or through a known or established target value (e.g. no species at risk of extinction, or 30% of marine waters designated in protected areas). A more detailed discussion of the considerations and process for selecting reference points is found in Samhouri *et al.* [5], and in Text S1 for Halpern *et al.* [2]. Our case study also used a spatio-temporal comparison, in which the present status of a goal for all Brazilian coastal states was compared to the best performing state over the analysis period. For example, the Tourism and Recreation goal uses as its reference value the highest score achieved across all states and all years of data available (i.e. Rio de Janeiro in 2011). A full description of the goal-specific reference points is provided in the Supporting Information and listed in Table S1 in Text S1.

The likely near-term future status of a goal, 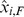, is given as:
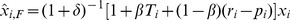
(4)


where *r*_i_ is Resilience, *p*_i_ is Pressures, and *T_i_* is the Trend. A discount rate (*δ*) was included in the equation, but was approximated to 0, because the likely future state is an assessment in the very near future [2]. Beta (*β*) represents the relative importance of the Trend versus the Resilience and Pressure terms in determining the likely trajectory of the goal status into the future. We assume β = 0.67 based on the idea that the direct measure of Trend is a better indicator of the near future than the indirect measures of Pressure and Resilience, and therefore carries twice the weight [2].

Trend is calculated as the change in Status (slope) over the previous five years. The annual rate of change was multiplied by five to give an estimation of the Status in the near-term future [2]. To calculate Pressures for each goal (*p_x_*) we evaluate both ecological (*p_E_*) and social pressures (*p_S_*). Ecological pressures are comprised of five broad categories: fishing pressure, habitat destruction, climate change, water pollution and species introductions. The contribution of individual pressures to the overall ecological pressure score is based on a weighting scheme having pressures ranked as ‘high’ (weight = 3), ‘medium’ (weight = 2), and ‘low’ (weight = 1) impacts on the goal, sub-goal, or component (see Table S7 in Text S1). Ecological pressures (*p_E_*) are calculated as the weighted-average of the Pressure categories relevant to each goal. Rankings were determined by literature review [2], modified slightly through expert judgment on Brazilian systems. Social pressures were based on a metric developed by The Economist Intelligence Unit (UIE; see Text S1), which ranks management effectiveness in Brazilian states. The UIE index is comprised of eight categories: Political Environment, Economic Environment, Tributary and Regulatory Environment, Policies for International Investment, Human Resources, Infrastructure, Innovation, and Sustainability determined at the state level. We used the aggregate score of all components of the UIE index for each coastal state.

The Pressure for each goal (*p_x_*) is therefore calculated as:
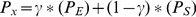
(5)


where γ is the relative weight for ecological vs. social pressures and is set equal to 0.5. Total Pressure scores range between 0 and 100 with 100 being the highest threat.

To calculate Resilience (Table S8 in Text S1) for each goal (*r_x_*) we assess three types of measures: ecological integrity (*Y_E_*), goal specific regulations aimed at addressing ecological pressures (*G*), and social integrity (*Y_S_*). When all three aspects are relevant to a goal, Resilience is calculated as:
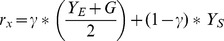
(6)


where the three measures are scaled 0–100, and gamma is assumed to be 0.5 (such that ecological and social Resilience components are equivalent). Ecological integrity (*Y_E_*) is measured as the relative condition of assessed marine species (see Text S1), regulations (*G*) are laws and institutional measures that support that goal and is calculated as the weighted average of those measures, and social integrity measures (*Y_S_*) is simply the UIE Index.

**Figure 1:**
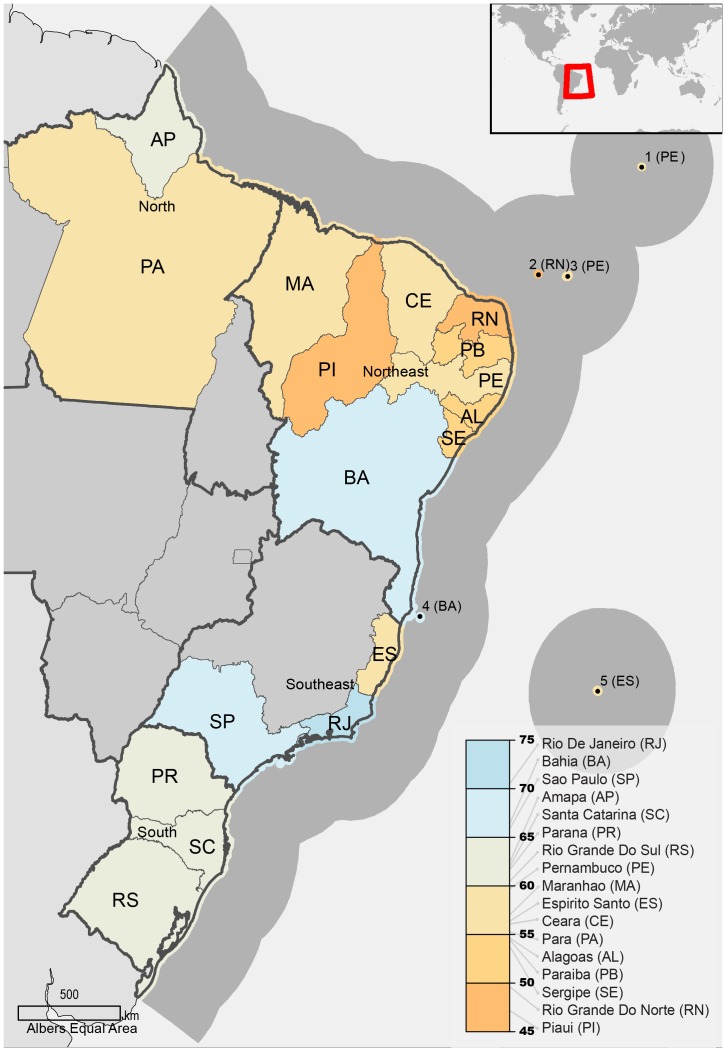
Brazil study region showing coastal states, colored by final OHI score and listed in legend by ranked score. The following islands were considered within the jurisdiction of states specified in parenthesis: 1. São Pedro & São Paulo Archipelago (PE), 2. Rocas Atoll (RN), 3. Fernando de Noronha (PE), 4. Abrolhos Archipelago (BA), 5. Trindade & Martim Vaz (ES).

The Index was calculated for each Brazilian coastal state (Figure 1) and for the entire country as an area-weighted average of coastal state scores. The scale of goal or sub-goal calculations was dependent on data resolution. Based on available input data and goal formulation, the spatial scale and geographic domain of analysis differed between goals (Table 2). When possible, we took advantage of state-level statistics such as tourism data, population census counts, and habitat data with direct relevance to the state's terrestrial coastline and coastal waters (0–12 nmi offshore). National level data included variables such as national level statistics, and data pertaining to the entire Brazilian EEZ (0–200 nmi). When only national data were available, the values for the goal's status and trends across states were identical and any variation in final score (Table 3) was due to the influence of pressure and resilience, which differed between states.

**Table 2:** Spatial resolution and geographic domain of goal calculations based on available input data and goal formulation.

Spatial resolution	Goal or sub-goal	Geographic Domain		
		Terrestrial coastline	Coastal waters (0–12 nmi)*	Federal waters (0–200 nmi)
Coastal State	Clean Waters	x	x	
	Tourism and Recreation	x		
	Mariculture (FP)	x	x	
	Lasting Special Places (SP)	x	x	
Mixed State and National	Carbon Storage	x	x	
	Coastal Protection	x	x	
	Habitats (BD)	x	x	
National	Fisheries (FP)			x
	Artisanal Opportunities			x
	Natural Products			x
	Livelihoods (LE)	x		x
	Economies (LE)	x		x
	Iconic Species (SP)			x
	Species (BD)			x

Where sub-goals are shown, the respective goal is indicated in brackets (for acronyms see Table 1).

**Table 3:** Overall Index, goal and sub-goal scores for Brazil (country) and each Brazilian coastal state.

Region		FP			AO	NP	CS	CP	LE			TR	SP			CW	BD		
	Index	FIS		MAR					LIV		ECO		ICO		LSP		HAB		SPP
Brazil	60	42	36	6	62	29	89	92	56	52	48	31	47	48	48	77	95	85	74
Alagoas (AL)	55	40	33	1	59	28	90	89	55	51	46	22	46	33	20	60	94	82	70
Amapá (AP)	62	42	42		62	28	93	94	54	50	46	3	47	73	98	90	96	85	74
Bahia (BA)	66	41	34	1	61	29	93	93	56	52	48	88	47	58	69	71	97	85	73
Ceará (CA)	56	41	36	12	60	29	75	76	55	51	47	34	47	35	24	85	90	81	73
Espírito Santo (ES)	57	42	35	3	61	29	95	94	56	52	48	15	47	38	28	62	97	85	74
Maranhão (MA)	57	40	34	0	60	28	87	88	55	50	46	9	46	53	60	79	93	82	72
Pará (PA)	55	41	34	0	60	28	92	93	55	50	46	1	46	37	29	74	96	84	72
Paraíba (PB)	55	40	33	1	59	28	87	89	55	51	46	11	46	44	43	62	93	82	71
Pernambuco (PE)	60	41	34	2	60	29	85	88	56	52	48	58	47	41	35	70	94	83	73
Piauí (PI)	47	40	33	1	59	27	81	82	54	50	45	2	45	27	10	31	91	80	69
Paraná (PR)	60	42	40	27	63	29	95	96	56	53	49	3	48	53	59	85	99	87	76
Rio De Janeiro (RJ)	71	44	36	0	65	30	99	99	57	54	50	100	50	57	65	77	99	88	78
Rio Grande Do Norte (RN)	50	40	34	5	59	28	33	74	55	50	46	33	46	32	17	79	77	74	71
Rio Grande Do Sul (RS)	60	43	36	0	63	30	100	100	57	53	49	5	49	42	35	84	100	88	77
Santa Catarina (SC)	62	42	46	66	62	29	93	94	56	52	49	37	48	39	31	77	99	87	75
Sergipe (SE)	54	40	34	2	60	28	89	90	55	51	47	11	46	45	45	47	95	83	71
São Paulo (SP)	66	45	37	1	66	30	97	97	58	54	51	29	51	63	75	95	99	89	80

Empty cells are goals not relevant to that region. Goals (two-letter codes) and sub-goals (three-letter codes) are reported separately; LE, SP and BD goals are the average of sub-goal scores; FP scores are the weighted average of sub-goal scores. Acronyms are the same as in Table 1.

To see how the Ocean Health Index compares with another across-sector index, we compared current versus likely future status scores for each state with an independent metric used in Brazil to track development status (FIRJAN Development Index score, IFDM). IFDM is an index of human development, measured in three areas: jobs and income, education, and health, providing a useful comparison to our evaluation of ocean health.

## Results

The overall Index score for Brazil was 60 out of 100, with state-level scores ranging from 47 to 71 (Table 3). Highest scoring goals were those relating to habitat condition, including Coastal Protection (score: 92) and Carbon Storage (89). The Biodiversity score for Brazil was 85, averaged across Habitats (95) and Species (74) sub-goals.

Mariculture (6) scored lowest in the Index at the national level. Other extractive goals or sub-goals, such as Natural Products (29) and Fisheries (42) were also low. Iconic species (47), scored lower than the Species sub-goal, indicating that a high proportion of culturally and aesthetically valued species are threatened (Table 3).

**Figure 2:**
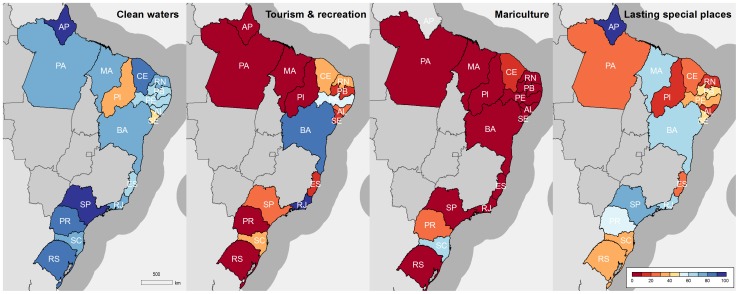
Goal and sub-goals calculated using state-level data.

Goals and sub-goals for which state-level data were used showed high variability among regions (Table 3, Figure 2). The most variable scores among states were for Tourism and Recreation, which ranged from a low of 1 in Pará to 100 in Rio de Janeiro. Similarly, Lasting Special Places ranged from 10 (Piauí) to 98 (Amapá), and the Mariculture sub-goal ranged from 0 (Maranhão, Pará, Rio de Janeiro and Rio Grande do Sul) to 66 (Santa Catarina). Clean Waters scored highest in Amapá (90) and São Paulo (95), and lowest in Piauí (31) and Sergipe (47).

**Figure 3:**
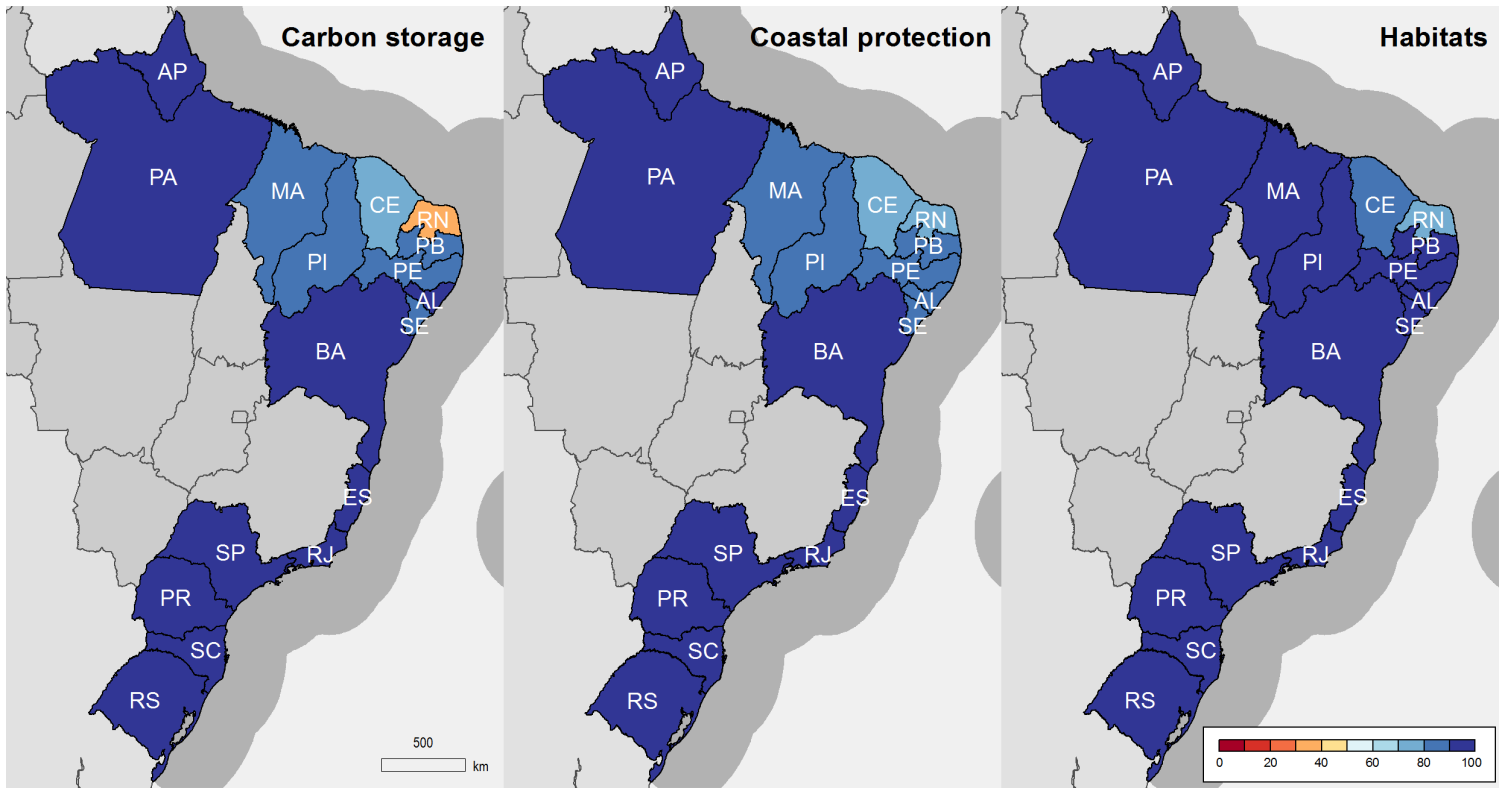
Goal and sub-goals using data from mixed national and state-level scales.

Scores for the Artisanal Opportunity goal (62) and Livelihoods (56) and Economies (48) sub-goals were low, but were evaluated at the country-level, likely masking important regional differences. Similarly, Carbon Storage (89), Coastal Protection (92) and the Habitats sub-goal of Biodiversity (95) showed high scores, with little variation among regions (Figure 3). Habitat data varied greatly in quality and quantity. Effects of these data constraints on the patterns we observed are discussed below.

**Figure 4:**
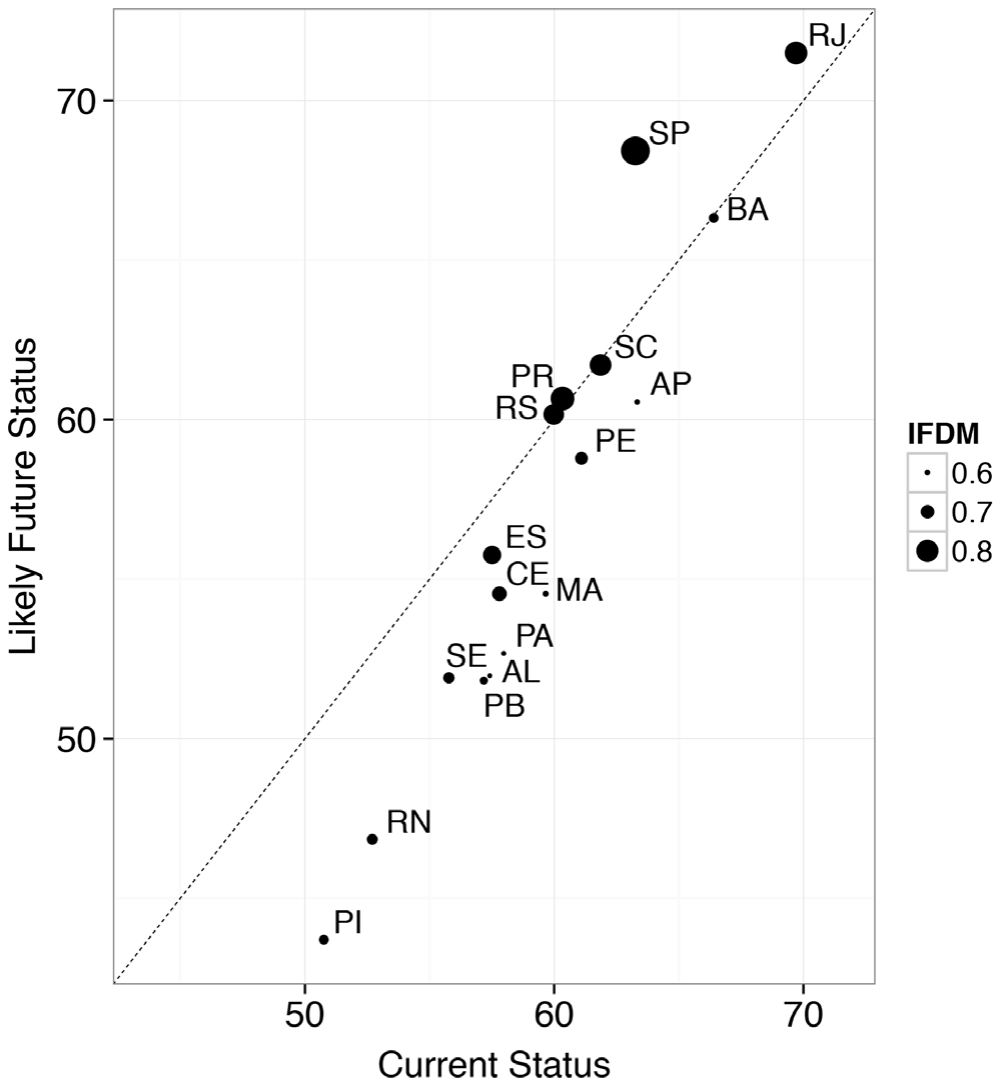
Current and likely future status for each state's overall Index score (axis values) and the value from an independent measure of development status (IFDM) used in Brazil (size of data point). Points below the dashed line are trending negatively into the future, and above are trending positively. IFDM scores range from 0 to 1 (low development =  0–0.4, average development  =  0.4–0.6, moderate development =  0.6–0.8, and high development =  0.8–1).

Comparisons of current and likely future status scores for each state's combined Index score revealed that the level of development of a state (assessed using the independent measure of development status, IFDM) was influential in determining the state's likely future score (Figure 4). The most developed states, São Paulo and Rio de Janeiro, are likely to improve their scores into the future. Paraná, Rio Grande do Sul, Santa Catarina and Bahia would likely maintain similar scores, while the remaining states are expected to have lower future scores.

## Discussion

This study is the first integrated assessment of the health of Brazil's ocean, based on the Ocean Health Index framework [2], and incorporating regional datasets. In the following sections we discuss sub-national patterns, lessons learned from this case study analysis, and key policy implications for Brazil.

### Spatial patterns

Differences in Food Provision scores between coastal states were driven by the Mariculture sub-goal (Table 3), as the Fisheries sub-goal was evaluated only at the national level. Despite the importance of wild-capture fisheries to coastal communities in Brazil, fisheries monitoring data is historically deficient, and it was not possible to determine state-specific landings. Mariculture scores were generally low due to low production (i.e. opportunity lost relative to potential production from mariculture) or production of unsustainable species. For example, whiteleg shrimp (*Litopenaeus vannamei*) was the most commonly cultivated species, with high production levels in the Northeast region, in particular Ceará and Rio Grande do Norte. However, these states did not achieve high Mariculture scores (Figure 2) due to the low sustainability of production for this species. Indeed, environmental and social problems associated with shrimp farming in Brazil are numerous, including severe mangrove loss, coastal erosion, pollution, land-use conflicts and loss of traditional livelihoods [7], [8]. Highest scores were achieved by Santa Catarina (score: 66) and Paraná (27), the two states with highest landings of bivalves relative to coastline length.

Habitat-based goals, including Carbon Storage, Coastal Protection and the Habitat sub-goal of Biodiversity, scored high across most states, with the exception of Rio Grande do Norte (Table 3, Figure 3) which has seen high rates of mangrove loss due to rapid expansion of shrimp farms (see Text S1). The high and relatively homogenous habitat-related scores are likely related to two reasons. The first is that for marine habitats such as coral reefs and seagrasses, there were challenges in obtaining data at the state-level, and historic reference points were not available within Brazil, such that Caribbean or South Atlantic averages were used. Such averages likely masked important localized declines, the result being remarkably similar scores between states (Figure 3). The second reason is related to Federal Law providing protection to mangrove and saltmarsh habitats. This situation may soon drastically change, as a recent revision of the Brazilian Forest Code legislation opens the possibility of using the salt flat portions of mangroves (locally known as “apicuns”) for mariculture, up to 10% in the Amazon Biome and 35% in the remaining coastal regions of the country (Brazilian Federal Law 12,651 of 2012).

The Lasting Special Places sub-goal was assessed using a national database of protected areas (including fully-protected and sustainable use designations at federal, state and municipal levels) and Indigenous lands. The remote state of Amapá achieved a score of 98, almost reaching the target value of 30% protection of the coastal zone (Table 3, Figure S1 in Text S1). Amapá contains the largest continuous extent of protected areas within the country in what is called the Biodiversity Corridor of Amapá. In the coastal zone, large areas have been set aside as fully protected and contain representative ecosystems of the Amazonian region, including the greatest extent of preserved mangroves in the Americas. Relatively high scores were also achieved by São Paulo (75), Bahia (69) and Rio de Janeiro (65) states, which contain a mosaic of areas, with a larger contribution of sustainable use areas and indigenous lands. States in the Northeast region of Brazil had the lowest scores, in particular those with small coastal areas (Table 3, Figure S1 in Text S1). Here, the majority of the population resides in urban areas along the coast, and even sustainable use areas are few. In our analysis, we chose not to include the category “Área de Proteção Ambiental” (APA), which typically comprise vast areas used for zoning multiple uses, not necessarily reflecting areas with specific protection (see Text S1). We note that a high Lasting Special Places score does not necessarily imply good biodiversity conservation, as this goal is driven by the cultural values people place on coastal areas (Table 1, Text S1).

The Tourism and Recreation goal had large variation in scores (Table 3, Figure 2), reflecting the variable importance of coastal tourism among regions. Rio de Janeiro (100), Bahia (88) and Pernambuco (58) had the highest scores as these states have a combination of high numbers and density of tourists (estimated by the density of coastal hotel employees per state; see Text S1), and are well known for the touristic attraction of their beaches and coastal cities. States in the North and South of Brazil had the lowest scores (with the exception of Santa Catarina).

Scores for the Clean Waters goal (Table 3, Figure 2) revealed that poorly developed states with low access to sanitation and waste management services, but low population densities (Amapá: 90), can score similarly to densely populated areas with good access to services (São Paulo: 95). However, only resident population density of coastal municipalities was used in estimating the Trash and Pathogen components of this goal. Many coastal urban areas receive a large influx of tourists in summer months, putting pressure on local infrastructure, including sanitation and waste management services [9], and likely polluting coastal waters, but these data are not available.

Perhaps unsurprisingly, a state's level of development influences its current and likely future status score. States with stronger economies, and better infrastructure and management, such as São Paulo and Rio de Janeiro, are more likely to pursue sustainable development paths and improve their Index scores, while less developed states show the opposite trend (Figure 4).

### Lessons Learned from Regional Application of the Index

Here we compare results from this case-study with scores for Brazil from the global analysis (year 2012; reported in Halpern *et al.* [2]) to help illustrate how regional applications of the Index may differ from global ones, and highlight what can be learned from efforts to conduct regional assessments. We note that some methodological changes and data updates affecting goal models have occurred recently and were used to calculate the global Ocean Health Index for year 2013; they also have been used to recalculate the Index for all countries for year 2012 (see oceanhealthindex.org/about/methods for details). However, we have chosen to focus on how results from the present case study relate to those from the initial, published assessment in Halpern *et al.* [2] as both share more methodological approaches. This allows a more direct comparison between global scores for Brazil and the results from this regional study, as differences are due to higher quality regional data, more direct measures of ocean health, and case-study specific model changes.

**Figure 5:**
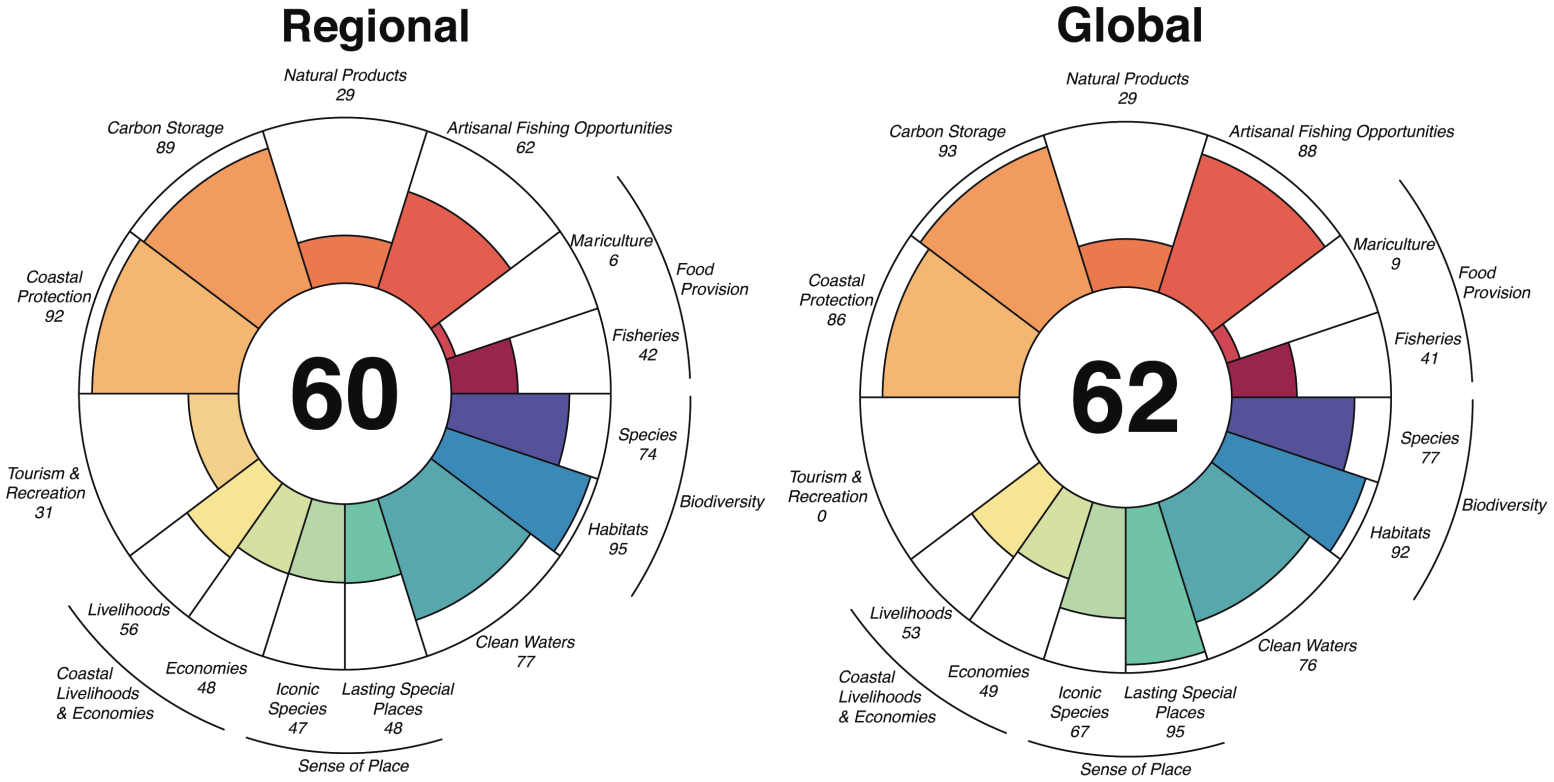
Goal and sub-goal scores for Brazil regional analysis (left), and Brazil global analysis (right). Key differences are found in Artisanal Fishing Opportunities, Tourism and Recreation, Lasting Special Places and Iconic Species. Overall Index scores (center) for the regional study are remarkably similar to global results for Brazil.

For some goals, the national scores remained similar to those from the global assessment [2], for example Mariculture and Clean Waters (Figure 5), even when significant model and/or data changes were made. As a consequence, the overall Index score for Brazil (60) was remarkably similar to the country score derived from the global analysis (62; Figure 5). These similarities suggest the Index may be able to broadly characterize ocean health even with poorer-quality, global-scale data.

The Index framework was designed to be flexible to different societal values and data contexts. Important model changes were made to some goals to reflect local conditions or to incorporate higher quality data. Below we discuss some changes to goal models and data layers and how they impact resulting scores.

The Food Production goal was analyzed using the same conceptual framework as in Halpern *et al.* [2], with some model adjustments (see Text S1). For the Mariculture sub-goal, we used national harvest statistics reported by each state, rather than country-level FAO statistics. Such data were not available for assessing the wild-capture component, the Fisheries sub-goal, of Food Production. For Mariculture, we were able to improve the estimation of the reference yield for each cultured species using historic time series of production; we also refined the total area of production available within each state (Text S1).

The Artisanal Opportunity goal scored lower than the global analysis (62 versus 88). The global model considered several aspects potentially related to the need and opportunity for people to fish artisanally, including regulations targeted to artisanal, subsistence and recreational fishing [2]. Until 2004, most fisheries in Brazil were considered “uncontrolled species” for which no management measures were in place [10]. For this reason, our model assumes that the opportunity to fish artisanally in Brazil is not limited by regulated access, but mainly by the condition of stocks. The lower Artisanal Opportunity score is indicative of the high proportion of stocks that fall within the overexploited, collapsed and rebuilding categories (for the most recent year (2006): 13%, 25% and 15%, respectively). The goal was calculated nationally, though significant regional differences likely exist. At present, standardized time series of small-scale fisheries landings are scarce, but would greatly increase the accuracy of this goal.

For Tourism and Recreation we used fine-scale data on hotel employment at the coastal municipality level, providing a better picture of ocean-related tourism than that derived from international arrivals data (used in Halpern *et al*. [2]). Data used here are of better quality and more comprehensive, as they also capture local participation in recreation. This altered the national aggregate score from 0 to 31 (Figure 5). The case-study approach has since helped to inform the global analysis, such that evaluation of the Tourism and Recreation goal in the updated assessment is now based on employment in the tourism sector, although without the benefit of higher spatial resolution data, as were available here (global data report the total number of jobs, not jobs within the coastal region).

For Biodiversity, regional assessments of threatened species showed significant differences from IUCN global assessments. For species assessed both globally and nationally, 58% held the same threat category, 33% had a higher risk of extinction, and 9% had a lower risk of extinction in national assessments. This difference was particularly notable for sharks and rays where 39% are considered threatened in Brazilian waters based on regional assessments [11] compared to only 17% based on global assessments [12]. Regional assessments, when available, were also used for the Iconic Species sub-goal of Sense of Place. The list of Iconic species was expanded (Table S6 in Text S1) to include a number of seabirds, in particular eight threatened species of albatross, and the Critically Endangered Atlantic goliath grouper (*Epinephelus itajara*). These changes and the inclusion of regional assessments led to a decrease in the Iconic Species score from 67 to 47 (Figure 5).

Clean Waters scores were nearly identical for both regional and global studies (77 and 76). The regional analysis used the same models for chemical and nutrient pollution [13], but modified the pathogens and trash pollution components to include datasets at the coastal municipality level, including: urban population densities, presence or absence of sewage treatment service (pathogens), and four types of waste management services (marine debris).

Finally, we adapted measures of pressures and resilience to address those that are important to the local context. We incorporated state-level data on ecological resilience (protected areas) and social resilience (UIE management index of Brazilian states). Pressure layers with new regional data sources included: Human Pathogens, Trash, Intertidal Habitat Destruction, and Shrimp farming in mangroves (See Text S1). The latter in particular was identified as an important aspect at the regional scale, with impacts on several goals (Table S7 in Text S1).

Our assessment shows that the Index can utilize data of varied quantity and quality. Our aim was to adapt to the regional context, while recognizing gaps and understanding potential limitations of the available information. We sought to gather the best currently available data that met minimum requirements for Index score calculation. For this, data needed to be collected with similar protocols across regions, available for all 17 coastal states (or sampled across all states, or ocean areas, but aggregated to the national level), and have enough spatial and/or temporal resolution for a reference point to be determined. When such requirements were not met, we used global data with country-level resolution. Better quality data sets were available for localized regions, which have been the focus of more intense research efforts in Brazil (e.g. Lagoa dos Patos region in Rio Grande do Sul state). Although such data are valuable for analyzing issues of a specific region, they are less suited for the integrated, comparative look used in the Index.

### Key Policy Implications

Our analysis reveals some important trends across states and at the country level. Here we focus on two key policy implications related to fisheries management and habitat protection.

First, Brazil has substantial room for improvement in sustainable food production. Landings from wild capture fisheries far exceeded sustainable target levels in the main portion of the Brazilian coast and Trindade and Martim Vaz islands (Figure S2 in Text S1). Similar results were found from fisheries assessments from a multi-year Brazilian research program called REVIZEE, with the majority of stocks either fully (23%) or over-exploited (33%) and little room for expansion into new fisheries [10]. Yet despite these clear indications of overexploitation, policy initiatives from the Ministry of Fisheries and Aquaculture have focused on increasing harvests. A new governmental initiative (Plano Safra) will invest 4.2 billion Reais (∼1.8 billion USD) in the fishing and aquaculture sectors with the goal of increasing total production to 2 million tons per year by 2014. Although the Mariculture scores suggest room for increased production in this sector, a greater focus on sustainable mariculture practices is needed if better ocean health is to be achieved.

Fisheries management in Brazil has been characterized by decades of open access to most fisheries and consequently to high fishing exploitation levels impacting both sustainability and profitability of its fisheries [10]. Brazilian policies for foreign fleets operating in the outer continental shelf and continental slope lack even minimal monitoring and enforcement, contributing to the decline of landings of many demersal stocks [14]. Substantial changes in current management of fisheries are needed, including implementation of a comprehensive and permanent monitoring system to evaluate stock status, and establishment of catch limits and other measures to protect and allow the rebuilding of marine resources, where needed.

The second set of policy implications relate to habitat-based goals. In our analysis, only 12% of the coastal zone (defined as 1 km inland and 3 nmi offshore) was in protected areas. These areas only cover 0.35% of the Brazilian EEZ (as noted above, APAs were excluded). This falls well below the target of 10% marine area to be protected by 2020 under the Convention of Biological Diversity [15]. A network of marine protected areas (MPAs) is important for protecting key habitats such as coral reefs [16], as well as other habitats not included in our analysis [17], [18]. For example, the largest contiguous rhodolith bed in the world has recently been mapped off eastern Brazil, and is estimated to account for 5% of the world's total carbonate banks, playing a significant role in carbon storage [19], but remains totally unprotected.

With an immense coastline and diverse coastal habitats, Brazil still lacks systematic mapping and monitoring data for its marine habitats. Although initiatives for broad-scale mapping and monitoring of marine and coastal habitats are emerging in Brazil (e.g. SISBiota: www.sisbiota.ufsc.br, and Rebentos: http://rebentos.org/), data from these projects were not yet available at the time of this study. For our analysis, we found that seagrass beds monitored with similar protocols currently exist for only 3 sites and coral reef data from repeated surveys were available for only 11 sites. Continuous, rather than sporadic or one-time monitoring of key sites for coastal and marine habitats is a priority. In the future it will be important to include habitats not yet incorporated in this study, such as seamounts, mesophotic and deep corals, and algae banks. More comprehensive and systematic mapping of marine habitats provides benchmarks that are useful to understand the effects of new developing activities and enables to detect in a timely manner the effects of potentially competing interests, such as oil development and offshore leasing occurring in Brazil, so as to regulate these activities based on management priorities.

While data are available for some economic sectors (e.g. tourism, mariculture, waste disposal, and protected areas), the country lacks monitoring plans for many of the types of information required to understand human uses of the marine environment, thus posing a practical challenge for long-term management of the health of marine ecosystems. Notwithstanding, we found that the Index can be a useful metric, using currently available information for illuminating ecological and social patterns related to ocean health. We also showed how it is a scalable, flexible approach that can be applied at different management units, and this flexibility will allow incorporating newer and more relevant data as these become available.

The results presented here represent a first attempt to assess ocean health in a comprehensive manner for Brazil. As such, this study offers an important baseline against which future change can be measured. It also highlights where better information is needed, and can help to guide policy and management actions at national and sub-national scales.

## Supporting Information

Text S1 Supporting Information.
(DOCX)
