# Supplementary material for: A Regional-Scale Ocean Health Index for Brazil
Source: PLoS One. 2014 Apr 2;9(4):e92589. doi: 10.1371/journal.pone.0092589 (PMC3973640; doi:10.1371/journal.pone.0092589)
Supplement: Text S1 — Supporting Information. (DOCX) [file pone.0092589.s001.docx]

# Text S1. Supporting Information

# 1. Study Area

We focused our analysis on Brazilian states directly adjacent to the ocean, such that each coastal state represents a reporting unit. Using state boundary data (on land), we define marine state waters using a fixed-distance 12 nautical mile (nmi) buffer from the shoreline, which we derive from a 1 km resolution global land-sea model [1]. This 12 nmi region represents approximately the area of coastal waters under jurisdiction of Brazilian coastal states. There were five island regions that occurred outside this 12 nm buffer. These were also included in the analysis and assigned to states as follows: Rocas Atoll (Rio Grande do Norte State), São Pedro and São Paulo Archipelago (Pernambuco State), Fernando de Noronha (Pernambuco State), Trindade and Martim Vaz (Espírito Santo State), and Abrolhos Archipelago (Bahia State).

**2. General Data Processing**

All spatial data use an Albers Equal Area Conic projection on the WGS84 datum, centered on 50^o^E, 25^o^S with parallels at 10^o^N and 40^o^S (registered online as SR-ORG:7390). During data integration, many data layers were natively in the South American Datum 1969 (SAD1969; EPSG:4618) so we re-project them using SAD_1969_To_WGS_1984_4 datum transformation parameters in ArcGIS 10 (ESRI 2010).

Whenever possible we used data available per state (or finer resolution). When data were not available at the state level, we used data for the entire Brazilian EEZ (0-200 nmi) and assigned values equally to all states.

To identify island regions to include in state-level assessments, we created a point layer using coordinates found by manually searching Google Earth. For our fixed-distance region calculations for these islands, we modified our land-sea model to ensure that at least a single land pixel was present for each island region. Both São Pedro and São Paulo Archipelago and Trindade and Martim Vaz add a significant amount of area, about 20 percent, to the Brazil EEZ adjacent to the mainland.

For large fixed-distance coastal buffers on land, we rasterized state boundaries at 1 km resolution, then created a 50 km buffer to cover the gaps from conflating the state boundaries and land-sea model data. For all other fixed-distance coastal buffers we buffered from the coastline derived from the land-sea model.

**3. Reference Points**

Reference points for calculating the status were chosen individually for each goal (Table S1), using the framework described in Samhouri *et al* [2]. For most goals, we maintained the same type of reference points as applied in the global analysis in Halpern *et al.* [1]. Details are given in the supplementary materials to Halpern *et al*. [1], and also briefly summarized in this section.

The benefits and challenges of establishing reference points are discussed at greater length within [2], however, the general approach was to choose reference points that follow SMART principles (Specific, Measurable, Ambitious, Realistic and Time-bound) [2-4]. Reference points may be described to fall within four classes: functional relationships, time series approaches, spatial reference points and societally-established targets.

The ideal approach is to use an empirical or theoretical functional relationship from which a reference point is calculated for the amount of benefit expected from the system. One example is provided by the concept in fisheries of maximum sustainable yield (MSY), which is the highest amount that may be harvested sustainably from a marine population. This can be used as a benchmark to assess the degree to which the goal of sustainable seafood provision is being achieved, and is generally derived from bioeconomic models which seek to capture the interaction of fish population and fishing fleet dynamics. However, such functional relationships may not be known for many places or goals. In these cases, other methods can be adopted such as spatial and time series comparisons [2], under the assumption that the status of the system at the point in time or the location chosen as reference is the condition to aspire to in order to fully satisfy the goal. Following [2], the historic benchmark is appropriate when the desired state occurred at a fixed time within the past (such as baseline habitat extents used in Carbon Storage and Coastal Protection goals, and in the Habitats sub-goal of Biodiversity), while a moving target is appropriate when the objective is to avoid declines based on a recent time window (e.g. avoid job loss within the last 5 years for the Livelihoods sub-goal of the Livelihoods and Economies goal). We also used societally established reference points for some goals or sub-goals when the management objective has been outlined, for example by international treaties or conventions. As an example, the reference point for the Iconic Species goal is to have all species listed as “Least Concern” under the IUCN Red List Criteria, which is in alignment with the United Nations Convention on Biological Diversity [5].

We modified the reference points for Mariculture and Tourism and Recreation status to combine both a spatial and temporal approach. Thus, each coastal state is evaluated based on the maximum benefit achieved across all states over the analysis time period. This improves on the simple spatial approach, i.e. only using values in the current year to select the benchmark, because even the state that achieved the benchmark value can receive a less-than perfect score if the highest value was achieved some time in the past and it is not performing well in the current year. The type of reference point for each goal is listed in Table S1, and described in detail in the goal model text below.

**Table S1. Type of reference point used for each goal and sub-goal. The asterisk indicates reference points that were altered from those presented in Halpern *et al.* [1].**

| **Goal** | **Sub-goal: component** | **Reference point type** |
| --- | --- | --- |
| Food Provision | Fisheries | Functional relationship |
|  | Mariculture | Spatio-temporal comparison* |
| Artisanal Fishing Opportunities |  | Established target* |
| Natural Products |  | Temporal comparison (historical benchmark) |
| Carbon Storage |  | Temporal comparison (historical benchmark) |
| Coastal Protection |  | Temporal comparison (historical benchmark) |
| Coastal Livelihoods and Economies | Livelihoods: jobs | Temporal comparison (moving target) |
|  | Livelihoods: wages | Spatial comparison |
|  | Economies | Temporal comparison (moving target) |
| Tourism and Recreation |  | Spatio-temporal comparison* |
| Sense of Place | Iconic Species | Known target |
|  | Lasting Special Places | Established target |
| Clean Waters |  | Known target |
| Biodiversity | Habitats | Temporal comparison (historical benchmark) |
|  | Species | Known target |

# 4. Goal- Specific Models

In the sections below we present an overview of the goal-specific models, with greater emphasis and detail on how models were changed for this regional study. Refer to Halpern *et al.* [1] for further detail on models used for the global analysis in 2012. Changes in methods used for the global analysis in 2013 and applied as a recalculation to 2012 can be accessed from: www.oceanhealthindex.org/about/methods.

## A. Food Provision

This goal model measures the amount of seafood sustainably harvested for human consumption, including wild or cultured stocks. Wild-caught landings for Brazil represent commercial fisheries from both industrial and artisanal small-scale sectors (see [6]). The target for this goal is to maximize the level of exploitation of local marine resources to produce food, relative to the ecosystem’s potential, and applies a penalty for the use of unsustainable fishing or aquaculture practices (where “sustainable practices” are defined as those that do not impair future harvest, regardless of their effect on other benefits such as biodiversity).

The goal is divided into two sub-goals: Fisheries and Mariculture.

**Fisheries sub-goal:**

The fisheries sub-goal was calculated following methods described elsewhere [1]. In brief, the sub-goal is calculated as the absolute difference between a country’s total landed biomass (B_T_) from the reference point of the multi-species maximum sustainable yield (mMSY). In the global model, the calculation includes a correction by a taxonomic reporting quality index (T_C_), which estimates the reliability of landings data by comparing them with the list of taxa reported to FAO (spatially allocated by the Sea Around Us Project) by neighboring countries that have a transboundary distribution. In the global study, where there was high heterogeneity in the reporting quality across countries, this was considered a proxy for management and used as a place-holder for a sustainability index (S_I_). Brazil comprises a large portion of the South Atlantic coast of South America, making comparisons to neighboring countries less informative. For this study, we assume that the reported catch is sufficiently representative and replace T_C_ with a catch-based sustainability index (S_I_), which evaluates the exploitation status of species caught within the country’s EEZ. The status of the fisheries sub-goal (X_F_) is calculated as:

$X_{F}=\left(1-\left|\frac{0.75*mMSY-B_{T}}{0.75*mMSY}\right|\right)*S_{I}$ (Eq. S1)

*S_I_* is calculated using the exploitation categories: Developing, Fully Exploited, Overexploited, Collapsed, and Rebuilding. These categories (Table S2) are modeled on those used by the FAO, and calculated using algorithms developed by the Sea Around Us Project, using, for each species, the current landings relative to the historical peak catch and the trend [7].

**Table S2. Definitions and weights assigned for each category of exploitations status.**

| **Exploitation Category** | **w** | **Definition** |
| --- | --- | --- |
| Developing | 1.0 | Stock landings have not reached a peak or peak occurs in the last year of the times series. |
| Fully exploited | 1.0 | Stock landings are between 50-100% of peak. |
| Overexploited | 0.5 | Stock landings are between 10-50% of peak. |
| Collapsed | 0.0 | Stock landings are < 10% of peak and recent trend is <0. |
| Rebuilding | 0.25 | Stock landings are between 10-50% of peak and recent trend is > 0. |

The sustainability index (S_I_) for each *t* year is then:

$S_{I,t}=\frac{\sum_{k=1}^{5} N_{k}*w_{k}}{\sum_{k=1}^{5} N_{k}}$ (Eq. S2)

where *N* is the number of species in each *k* category of exploitation and *w* is the weight assigned to each category of exploitation status.

We note there has been recent scientific discussion [8,9] over the reliability of the catch-based model used in Halpern *et al.* [1] and applied here. We recognize there are inherent limitations to catch-based approaches, but argue that assessment of data-poor fisheries (comprising ~80% of fisheries biomass and the majority of stocks in most countries) requires use of catch-based methods. This holds true in Brazil, where only a handful of stock assessments have been carried out. The FAO produces a regular report of the state of the world fisheries, including stock status assessments for a small proportion of the total number of species reported in their international statistics, that calls for a better, more comprehensive way to model data-poor stocks [10], and there has been recent impetus to develop and improve such models in the scientific literature [11-14]. Given the lack of information required to implement formal stock assessments, and that those stocks that are formally assessed are not necessarily representative of the status of unassessed ones, we argue that it is better to use a data-poor approach than none at all [7, 12, 15].

The trend was calculated from the slope of the Status scores over the most recent five years of data (2001-2006). The pressures to this goal are the same as the global model, with new data used for Habitat Destruction in the Intertidal area, and with an added pressure from shrimp farm expansion (see Data Layers). Ecological resilience measures for this goal follow Halpern *et al.* [1]. Social resilience uses the UIE Government Effectiveness Index for Brazilian states.

**Mariculture sub-goal:**

The mariculture sub-goal was calculated at the state-level for Brazil using harvest data reported by the Brazilian Institute of the Environment and Renewable Natural Resources (IBAMA) for years 2001-2007. The data are for marine aquaculture species only. Reporting of finfish and scallops were sporadic, with only a few states reporting low level harvests (typically less than 15 tons) in some years. For this reason, they were excluded from the analysis.

The resulting species evaluated for Brazil were: South American rock mussel, also known as brown mussel (*Perna perna*), cupped oysters, and white-legged shrimp (*Litopenaeus vannamei*).

The mariculture status (X_M_) for state *i* in year *t* is the harvest-weighted score for each species *k* produced in that state, such that:

$X_{M(i,t)}=\sum_{k} w_{i,k,t}x_{M(i,k,t)}$ (Eq. S3)

where:

$w_{i,k,t}=\frac{Y_{i,k,t}*S_{M,k}}{\sum_{k} (Y_{i,k,t}*S_{M,k})}$ (Eq. S4)

$x_{M(i,k,t)}=\frac{Y_{i,k,t}}{R_{k}*A_{i,k}}*S_{M,k}$ (Eq. S5)

For each species *k* within the state, the score was determined by the yield (Y_i,k,t_), the reference sustainable production per unit area (R_k_) and the total potential farming area (A_i,k_). The sustainability score (S_M,k_) is based on part of the Mariculture Sustainability Index (MSI; [16]) and adjusts the credit given to each species’ harvest based on the sustainability of production, intended as the ability to maintain production in the longer term (Table S3). The sub-indices we chose to use from the MSI evaluate the treatment of wastewater, the origin of the feed (i.e. fishmeal or other), and the origin of seed (i.e. hatchery or wild caught), see Halpern *et al.* [1] for details. The species scores are then combined at the state level based on relative sustainable yield (*w_i,k,t_*).

The reference sustainable production (R_k_) was the maximum adjusted production (i.e. corrected by the sustainability score) achieved per unit area for each species across all states. This assumes that any state, given the appropriate cultivation methods can achieve a similar yield per area. For mussels and oysters, the reference point was calculated as the maximum production of Santa Catarina state (the highest producer) over the time series, normalized by its coastal area (1 km coastal strip), as the exact area occupied for production of these species was not available. For shrimp we were able to determine the maximum production per square kilometer of shrimp farms using data on the production and extent of farms for states in 2000 (see Data Layers below). The reference values used are shown in Table S3.

**Table S3. Mariculture sustainability scores and reference production values for species produced in Brazil.**

| **Species (k)** | **Sustainability Score (S_M,k_)** | **Reference Production (R_k_; tonnes/km^2^)** |
| --- | --- | --- |
| Whiteleg shrimp | 0.30 | 505.09 |
| South American rock mussel | 0.81 | 16.52 |
| Cupped oysters nei | 0.90 | 4.26 |

The total potential farming area (A_i,k_) for mussels and oysters was simply the coastal strip of each state (we did not discount areas of high coastal urbanization). For shrimp, the potential farming area was calculated as the sum of existing shrimp farms in 2010, plus the available area of unprotected mangroves. This is meant to reflect the current reality of expansion of shrimp farming operations within several states in Brazil [17].

States are evaluated based on the species they are currently producing, and are not penalized for the lack of production of a species that could in theory be cultivated. For example, though Rio de Janeiro state has areas of unprotected mangrove, it is not currently a producer of shrimp, and the score for shrimp is therefore not computed.

The trend in Mariculture was calculated as the slope of the status scores in the past five years (2002-2007). Following Halpern *et al.* [1], pressures used for this goal include pollution-related ecological pressures (Table S7) and resilience included a number of measures directed at improved aquaculture practices (Table S8).

**Combining sub-goals:**

The two sub-goals were combined into a single goal score via a proportional yield-weighted average:

$X_{FP}=\frac{B_{T}}{Y_{T}}*X_{F}+\frac{Y_{mar}}{Y_{T}}*X_{M}$ (Eq. S6)

where B_T_ is the wild-caught fishing yield for Brazil in the most recent year (2006), and Y_mar_ is the total mariculture yield summed across all Brazilian coastal states in the most recent year (2007), and Y_T_ is the total yield (sum of *B_T_* and Y_mar_). The relative contribution of the subgoals to the score is thus weighted based on their current contribution to seafood production.

## B. Artisanal Fishing Opportunities

Artisanal Fishing, also known as small-scale fishing, is the most important marine extractive activity in Brazil, involving at least 940,000 people who are registered fishers [18], with an unknown number of additional fishers and processors who directly depend on this activity for their livelihoods. It provides not only a source of food and income, but is part of the cultural identity of those involved. Artisanal fishing accounts for about 60% of total fish landings in Brazil, and 70% in regions such as the Brazilian northeast [19]. These landings are captured in the fisheries sub-goal above. In this goal we measure the opportunity to engage in the practice of artisanal fishing for cultural and/or economic purposes.

In the global model [1] this was assessed as a function of the need (assessed using poverty indicators), and the accessibility (assessed through institutional measures that support small-scale fishing), with a place-holder for stock status (which could not be assessed at global scales for artisanal-scale fishing). For Brazil we consider that the primary driver of artisanal opportunity is the availability of fish to capture (i.e. the condition of the stocks). Because the scale of analysis for which we had stock status information was national, we chose not to include a measure of artisanal need (levels of poverty), which has great variation within Brazil. In addition, we assume that access to fishing is largely open because permitting and regulations from the Ministry of Fisheries are not considered restrictive, and in most cases, neither is physical access.

The Status for this goal (X_AO_) is therefore measured simply as:

$X_{AO}=S_{I}$ (Eq. S7)

where S_I_ is a sustainability index calculated using the exploitation status of species (see Fisheries sub-goal of Food Provision). The reference point for this goal is an established target of 1.0, that is, all stocks are categorized as either Developing or Fully Exploited. Due to the widespread nature of artisanal fisheries throughout Brazil, and the major contribution of small-scale activities to total landings for the country, all species were considered possible targets of artisanal fishing activities.

The Trend was calculated as the slope of the status scores over the past five years (2001-2006). The pressures to this goal are the same as the global model, with new data used for Habitat Destruction in the Intertidal, and with an added pressure from shrimp farm expansion (see Data Layers). Ecological resilience measures for this goal follow Halpern *et al.* [1]; social resilience uses the UIE Government Effectiveness Index for Brazilian states (Tables S7 and S8).

The model is currently calculated at the national-scale, and the same score is assigned to all coastal states. Slight variation between states is due to the effect of pressures and resilience on goal scores. Assessment of this goal could be greatly improved if reliable state-level landings data were available.

## C. Natural Products

This goal measures the ability to maximize the sustainable harvest of non-food natural products from marine sources. These products do not include bioprospecting, which focuses on potential value rather than current realized value, or non-living products such as oil, gas or mining products, which by definition are not sustainable (see [1]).

For Brazil, we had FAO export data at the national scale for: fish oils, ornamental fish, seaweeds and sponges. Coral data, though reported in the statistics for Brazil from 1994 to 2008 were removed from the analyses as coral trade of any species is now banned (IBAMA Lei de Crimes Ambientais n.9605, February 1998, Article 33).

As in the global assessment [1], the status of each product was determined by the most recent harvest rate (in metric tons) relative to the maximum value (in 2008 USD) ever achieved, under the assumption that the maximum achieved at any point in time was likely the maximum possible. Thus, we use a historical benchmark as our reference point (Table S1). We created a buffer around this reference point such that any value within 35% of the peak was set to 1.0 and values below this were rescaled. Harvest scores were then adjusted by estimates of the sustainability of the harvest rate for each type of product. This sustainability term is calculated using the log-transformed intensity of harvest per km^2^ of coral and/or rocky reef, depending on the product (this is termed the “exposure”). For fish oils, the exposure was calculated using stock status assessments, as described in sections for Food Provision and Artisanal Opportunity. The viability of the harvest was also assessed for ornamental fish through an estimate of the sustainability of the practice.

The status of each *i* natural product (X_NP, i_) was therefore:

$X_{NP,i}=H_{p,i}*S_{p,i}$ (Eq. S8)

where *H_p,i_* is the harvest level for a product relative to its own (buffered) peak reference point, and *S_p,i_* is the sustainability of that harvest, with:

$S_{p,i}=1-\frac{E+V}{N}$ (Eq. S9)

where *E* is the exposure term, *V* is the viability term and *N*=1 or 2, depending on whether or not a viability term is used.

The Status score for each product was calculated for the five previous years, and the slope of this was used to calculate the Trend for each product. Pressures and Resilience measures were assigned to each product following Table S7 and Table S8. An Index score was calculated for each product, and combined into a single Natural Products score (X_NP_) using the weighted average of the individual product scores, such that:

$X_{NP}=\frac{\sum_{p=1}^{N} w_{p}x_{p}}{N}$ (Eq. S10)

where *N* is the number of products that have ever been harvested and *w_p_* is the proportional peak dollar value of each product relative to the total peak dollar value of all products (in 2008 USD).

## D. Carbon Storage

This goal measures carbon storage and sequestration in coastal habitats, focusing on three habitats that are known to provide meaningful amounts of carbon storage: mangroves, seagrasses, and salt marshes.

The Status of the Carbon Storage goal (X_CS_) was measured as a function of each habitat’s current condition (C_c_) relative to a reference condition (C_r_), a variable that weights the relative contribution of each habitat type (k) to total carbon storage measured as the amount of area each habitat covers (A_k_) relative to the total area covered by all three habitats (A_T_). We assumed similar carbon sequestration rates and storage capacity for all three habitats (see [1]).

The reference condition (C_r_) was determined specifically for each habitat type. For salt marshes the reference year is 1975 (see [20]). For mangroves, we knew the current (2010) extent per state [21], but only had a total country extent for the reference year (1980) [22]. We apportioned the total reported mangrove extent for Brazil in 1980 by state using a linear regression model that estimates the percent of mangrove loss per state. This model uses the proportion of mangrove area per state known for 2010 and time-series data on changes in coastal urban population density and shrimp farm development (see Data Layers). Data to assess current and reference condition for seagrasses did not meet minimum data requirements. Data were available only for three sites in Brazil within the time period 2002-2010 (no data for a reference condition). For this reason, we used available data from adjacent EEZs (countries in the South Atlantic) and used georegional averages as current condition (C_c_) and reference condition (C_r_) values for Brazil. A linear model was fitted to the data for all countries, and the mean of predicted values for 1979-1981 was used as the reference condition, and the mean of predicted values of the three most recent years (2008, 2009, 2010) was used as the current condition.

The Status is calculated as:

$X_{CS}=\sum_{1}^{k} \left(\frac{C_{c}}{C_{r}}*\frac{A_{k}}{A_{T}}\right)$ (Eq. S11)

where A_T_ is simply the sum of the total known area for each habitat type measured within the state (see Coastal Protection):

$A_{T}=\sum_{i=1}^{k} A_{k}$ (Eq. S12)

Trend for this goal is the slope of the change in Status over the past 5 years. See Biodiversity goal (section J) for details on trend calculation for each habitat. Ecological pressures and resilience varied by habitat, but social pressures and resilience were assumed to affect all habitats equally (Table S7, and Table S8).

## E. Coastal Protection

This goal model aims to assess the amount of protection provided by marine and coastal habitats to coastal areas that people value, both inhabited and uninhabited [1]. Although other habitats may provide protection to coastal areas, such as sand dunes, the ones for which we had data are mangroves, coral reefs, seagrasses, and salt marshes.

The Status of this goal was calculated as a function of the amount and/or condition of marine habitat(s) relative to their reference states and the ranked protective ability of each habitat type, such that:

$X_{CP}=\sum_{i=1}^{k} \left(\frac{C_{c}}{C_{r}}*\frac{w_{k}}{w_{\max}}*\frac{A_{k}}{A_{T}}\right)$ (Eq. S13)

where C is the condition at current (c) and reference (r) time points, and w is the rank weight of protective ability, and A is the area within each states 12 nmi jurisdiction boundary for each *k* habitat type, proportional to either the maximum (*max*) ranks of any habitats present or total (T) current amounts of all protective habitats (Equation 13). For mangroves we focused only on the most coastal portion of mangrove forests as they are the main source of coastal protection. We used a perimeter measurement (30m cell size) to count the area of mangrove extent anywhere within the fixed-distance buffers for 200 nmi offshore and 50 km inland. For seagrasses we used the total reported extent of seagrasses in Brazil [23] divided by the coastal area of each state. For coral reefs we calculated the extent per coastal waters of each state using maps of coral reef distribution (500 m resolution) from Reefs at Risk [24]. The salt marsh extents for Santa Catarina and Rio Grande do Sul states are from national statistics [21].

To calculate the reference state for coral reef status within Brazil we lacked a minimum of two data points within the time period 1980-1995 (which was considered the acceptable range to use as “reference” years). We therefore estimated the status as the averages of scores from 24 countries within the Caribbean ecoregion that had sufficient coral data (see Halpern *et al*.[1]) and Selig *et al.* [25]) . For each of those countries, we fitted a linear model to the data available, pooled across all sampled sites, and we defined the ‘current’ condition (health) as the mean of the predicted values for 2008-2010, and the reference condition as the mean of the predicted values for 1985-1987.

Rank weights for the protective ability of each habitat (w_k_) come from previous work [26] that ranks mangroves and corals as 4, salt marshes as 3, and seagrasses as 1 (higher values are better).

The Trend for this goal is the annual change in ranked condition weighted (averaged) according to the relative proportion of the habitat (A_k_/A_T_) and then converted to a 5-year time horizon. See Biodiversity goal (section J) for details on trend calculation for each habitat. Ecological Pressures and Resilience varied by habitat, but social Pressures and Resilience were assumed to affect all habitats equally (see Table S7, Table S8).

## F. Tourism and Recreation

This goal model aims to assess the value that people have in experiencing and enjoying coastal areas. The model developed for the global Ocean Health Index [1] was changed to use information on hotel employees for each coastal municipality in Brazil.

We measured the Status of this goal (*X*_TR_) for each coastal state as the density of hotel jobs in coastal areas, such that:

$X_{TR}=\frac{Jobs_{hotel}}{\log(A_{coast})}*S_{t}$ (Eq. S14)

where Jobs_hotel_ is the sum of all hotel jobs within coastal municipalities of the state, A_coast_ is the state’s coastal strip (1 km inland buffer), and S_t_ is a sustainability factor for each year for Brazil from the Travel and Tourism Competitiveness Index (TTCI).

This model formulation assumes that the majority of coastal hotels are located in proximity to the shoreline, and that the number of hotel employees is directly proportional to the volume of tourists an area receives. A report evaluating drivers of tourism in Brazil found a significant positive relationship between number of tourists and number of hotels [27]; here we incorporate hotel employees which is likely a more sensitive metric, given that hotels can vary greatly in size and economic changes are likely to be reflected more quickly in number of jobs than number of hotel establishments.

We log-transformed coastal area under the assumption that density of hotel employees is not necessarily a measure of sustainable tourism, but that a balance likely exists between the density of tourists and the absolute number of tourists that a state receives (even if spread out over a larger area). We explored other models in which coastal area was not log-transformed and found that only states with small coastal areas scored high. As we do not have a reference value for what optimal tourism density (measured through hotel employment density) would be, we log-transform coastal area so that states with small coastline are not overly favored and states with large coastline are not penalized.

Although the TTCI was included in the model as a measure of sustainability, this value is calculated at the national level, and was therefore applied equally to all states. Due to this, it has no effect on differences between regions within Brazil. The model could be improved if a state-specific index assessing sustainability of coastal tourism were available.

The reference value used was the highest Status value across all states over the time series, which was Rio de Janeiro in 2011. The Trend was calculated as the slope of the average Status scores for 2006-2011.

Pressures to this goal are from pollution as in the global model ([1], Table S7). Resilience measures come from the UIE Government Effectiveness Index for Brazilian states, and from the CBD questions targeted at water pollution (Table S8).

## G. Coastal Livelihoods and Economies

This goal is to maintain (i.e. avoid the loss of) coastal and ocean-dependent livelihoods (jobs) and productive coastal economies (revenues), while also maximizing livelihood quality (relative wages).

This goal was modeled and used the same data sets as the global study [1] because no higher resolution data were found for Brazil. Income data were updated to include wage data until 2008 ([www.data.nber.org/oww](http://data.nber.org/oww)). Briefly, the goal is composed of two equally important sub-goals, Livelihoods (L) and Economies (E), which are assessed across several marine-related sectors. The full list of which sectors had available data for each sub-goal are shown in Table S4.

**Table S4. Sectors for which data were available for Brazil for each of the three measures for Coastal Livelihoods and Economies.**

| **Sector** | **Jobs data** | **Wages data** | **Revenue data** |
| --- | --- | --- | --- |
| Tourism | x | x | x |
| Commercial fishing | x | x | x |
| Oil and gas |  | x |  |
| Marine mammal watching | x |  | x |
| Aquarium fishing |  |  | x |
| Mariculture |  |  | x |
| Shipping and Transport |  | x |  |
| Ports and harbors |  | x |  |

Livelihoods includes two equally important sub-components, the number of jobs (*j*), which is a proxy for livelihood quantity, and the per capita average annual wages (*w*), which is a proxy for job quality. Economies is composed of a single component, revenue (*e*), measured in 2010 USD.

There is no established target number of jobs or amount of revenue, but the objective is generally to suffer no net loss of each, at least using the recent past as a reference, so these two sub-goals employ a moving baseline reference point. The two metrics (*j*, *e*) are calculated as the relative value in the current year (or most recent available year), *c*, divided by a moving reference period, *r*, defined as 5 years prior to *c*. The two parameters are corrected for overall economic patterns, so as to capture whether changes occurred specifically in marine-related sectors (i.e. exclude the effects of economic trends that are not linked to the marine environment).

The Status of this goal (X_LE_) is the average of the Status of the two sub-goals:

$X_{LE}=(X_{L}+X_{E})/2$ (Eq. S15)

The Livelihoods sub-goal (X_L_) is measured as:

$X_{L}=\frac{\left(\frac{\sum_{1}^{k} j_{c,k}}{\sum_{1}^{k} j_{r,k}}+\frac{\sum_{1}^{k} g_{B,k}}{\sum_{1}^{k} g_{r,k}}\right)}{2}$ (Eq. S16)

where *j* is the adjusted number of direct and indirect jobs within sector *k* within Brazil and *g* is the average PPP-adjusted wages per job within sector *k*. Jobs are summed across sectors and measured at current (*c*) and reference (*r*) time points. Wages are averaged across sectors within Brazil (*B*), and compared to a reference country (*r*) with the highest average wages across all sectors. Refer to Halpern *et al.* [1] for further detail.

The Status of the Economies sub-goal (*X_E_*) is assessed as:

$X_{E}=\sum_{1}^{k} \frac{e_{c,k}}{e_{r,k}}$ (Eq. S17)

where *e* is the total adjusted revenue generated directly and indirectly from sector *k*, at current (*c*) and reference (*r*) time points. Refer to Halpern *et al.* [1] for further detail.

The target value for the Status of this goal is to be equal to or greater than 1.0, but scores were capped at the maximum score of 1.0.

The Trend was calculated as the slope in the individual sector values (not summed sectors) for *j*, *w*, and *e* over the most recent five years, corrected by national trends in employment, average wages, and GDP, respectively. We then calculated the average trend for jobs across all sectors, with the average weighted by the number of jobs in each sector. We calculated the average trend for wages across all sectors. We calculated the average for revenue by averaging slopes across sectors weighted by the revenue in each sector. We then averaged the wages and jobs average slopes to get a Trend value for Livelihoods (X_L_) and the weighted average slope for revenue is the Trend value for Economies (X_E_).

For ecological pressures we evaluated the potential stressors to each sector and then used the average weight across all the sectors as the multiplier for each stressor intensity value (see [1]). For the social pressures, we used three measures that influence social integrity: social evenness (SE), the Global Competitiveness Index (GCI), and the UIE Government Effectiveness Index for Brazilian states (UIE) (Table S7). Social pressures were calculated as: [(1-SectorEveness)+(1-UIE)+(1-GCI)]/3 for the Livelihoods sub-component and [(1-WGI)+(1-GCI)]/2 for the Economies sub-component. The overall Pressures score was then the average of the ecological and social pressure scores. For Resilience, we used (SE +UIE+GCI)/3 for Livelihoods and (UIE+GCI)/2 for Economies.

## H. Sense of Place

This goal captures the value of coastal and marine systems as part of people’s cultural identity. The goal is divided into two sub-goals: Iconic Species and Lasting Special Places, which are weighted equally when combined to create a single goal score. The Iconic Species sub-goal was calculated at the national level and the same score assigned to all coastal states. The Lasting Special Places sub-goal was calculated by state.

**Iconic Species sub-goal**

Iconic species are those that are viewed as important to society because of their existence or aesthetic value, or association with traditional activities. Habitat-forming species are not included in this definition of iconic species, neither are species that are valued mainly for their extraction (e.g. commercially fished species), although these may be important to a sector or individual.

The list for Brazil was comprised of the original list used in the global analysis [1], which include priority species and flagship species defined by the World Wildlife Fund that occur in Brazil, and a list of species that are the focus of governmental or non-governmental conservation projects in Brazil (see Data Layers below).

The Status for this sub-goal (X_I_) is the percent of iconic species of Least Concern status (as defined by the IUCN Red List), such that:

$X_{I}=\left(\frac{\sum_{i=1}^{N} w_{i}}{N}\right)*100$ (Eq. S18)

where *w_i_* is the status weight assigned to each threat category (Extinct, Critically Endangered, Endangered, Vulnerable, Near Threatened, and Least Concern, see Table S5), and *N* is the number of iconic species in the region. This formulation gives partial credit to species that exist, but are in one of the other threat categories. The reference point is to have the risk status of all species as Least Concern. Species that have not been assessed or are in the IUCN category Data Deficient are not included in the calculation.

This sub-goal was calculated at the national level for Brazil because we assume that the presence of a species within Brazilian waters is sufficient to make it Iconic throughout all states.

The IUCN provides information on whether assessed species are increasing, stable, or decreasing in population size or whether the trend is unknown. The Trend calculation for this goal is based on the average of the recorded categorical trends (excluding unknown trends) for all iconic species in Brazil that have been assessed by IUCN. Scores are: 0.5 (increasing population), 0.0 (stable), -0.5 (decreasing population).

Pressures to this goal are shown in Table S7. For Resilience measures we used being a signatory on a number of conventions and treaties as ecological resilience, and the UIE Governance Effectiveness Ranking as a social resilience measure (Table S8).

**Lasting Special Places sub-goal**

This sub-goal focuses on the geographic locations that hold particular value for aesthetic, spiritual, cultural, recreational, or existence reasons. Because it is difficult to identify special places, we assume that areas that are protected represent special places. In addition, we establish a target reference value of 30% of coastline and coastal waters that should receive some level of protection (see [1]).

The Status of this sub-goal is calculated using:

**$X_{LSP}=\frac{\left(\frac{\%_{CMPA}}{\%_{ref\_CMPA}}+\frac{\%_{CP}}{\%_{ref\_CP}}\right)}{2}$**  (Eq. S19)

where CMPA=coastal marine protected area, CP= coastline protected, and Ref= 30% for both measures. For coastal waters we used a 3 nmi buffer from shore, and for coastlines we used the first 1km wide strip of land inshore.

Data on protected areas were obtained from the Brazilian Ministry of the Environment’s database on the national system of protected areas (CNUC: Cadastro Nacional de Unidades de Conservação). We used municipal, state, and federal protected areas regardless of the level of protection, however, we excluded the category “Área de Proteção Ambiental” (APA) because this category comprises large areas in some states not necessarily created in special places, but as multiple use management tools. We recognize there may be some exceptions, but overall, the inclusion of APAs would inflate scores for several states. We also included Indigenous Lands, which receive a formal designation that is different from the categories under the protected areas system, but is also recognized as a special place for their cultural importance.

To calculate the Trend, we calculate annual percentage increase in area for 2005-2010, using year of designation. In our analysis we assume that designated areas cannot be un-designated, such that the Trend is positive. Pressures to this goal derive from pollution and habitat destruction (see Table S7). Resilience measures come from CBD questions relating to pollution, habitat destruction and amount of money (proportional to GDP) invested in protected areas, as well as the UIE Governance Effectiveness Ranking (Table S8).

## I. Clean Waters

People value marine waters that are free of pollution and debris for aesthetic and health reasons. This goal evaluates the impact of pollution from four components that compromise Clean Waters: eutrophication (nutrients), chemicals, pathogens, and marine debris. The Status of these components is the inverse of their intensity (i.e. high input is a low Status). The goal scores highest when the contamination level is zero.

The data used to model the components for eutrophication (nutrients) and chemicals was the same as the global assessment [1, 28]. In short, nutrient pollution is estimated using a model of land-based nitrogen inputs and chemical pollution is measure via three global datasets on pollution from agricultural pesticide use, runoff from impervious surfaces, and commercial shipping and ports.

The data to characterize pathogen and marine debris pollution were developed for this case study using state-level data for Brazil. We used the same approach to model both components, namely the number of people in coastal areas without access to sewage treatment (pathogens), and without access to improved solid waste management of three types (marine debris).

To estimate pathogen intensity we multiplied the average population density of all coastal municipalities within the state by the percentage of the population within the coastal municipalities without access to sewage treatment. Data on the presence or absence of sewage treatment services for all coastal municipalities were obtained from the Brazilian Institute of Geography and Statistics (IBGE, data from 2008). This allows states within Brazil that have low coastal population densities and low access to improved sanitation to score better than states with high population density and improved access if the absolute number of people without access is lower.

To calculate marine debris intensity we used data on the presence or absence of four types of solid waste management in each coastal municipality, namely: access to beach clean-up services, household garbage collection, household recycling collection, and garbage collection in public streets (data from IBGE for 2008). The assumption is that the presence of these services will decrease the input of marine debris in coastal waters. For each coastal municipality, a combined score for improved solid waste management was calculated such that half the score was determined by the presence of beach clean-up service and the remaining half by the presence of each of the other three services. Greater weight was given to beach clean-up service as it is more directly related to impacts on coastal waters. If all services were present, the coastal municipality received a 1.0. To calculate the marine debris score for each state we multiplied the average score for solid waste management across all coastal municipalities by the population density score.

The Status for this goal (X_CW_) is then calculated as the geometric mean of the four components, such that:

 (Eq. S20)

where *a*= 1- the number of people without access to sewage treatment (i.e. pathogen input), *u* = 1- (nutrient input), *l* = 1- (chemical input), and *d*=1-(marine debris input). The goal is calculated only for coastal waters (3 nmi offshore) for each coastal state in Brazil.

For Trend calculation of nutrients and chemicals, we used a time series of FAO data for Brazil on tonnage of fertilizers and pesticides consumed (see [1]). The Trend for pathogens used a global dataset on access to improved sanitation (World Health Organization and United Nations Children’s Fund, Joint Monitoring Programme, [www.wssinfo.org](http://www.wssinfo.org)), which for Brazil increased from 72% in 1995 to 80% in 2008, as a discount factor on current access to sewage treatment (IBGE data for 2008). For marine debris the Trend was calculated as the slope between predicted values in 2005 and 2010 from a linear model of coastal population density (R^2^=0.90+/-0.25), aggregated from coastal municipalities in 1991, 2000, and 2010. The Trend for the Clean Waters goal was then calculated as the average trend for each of the pollution sub-components.

This goal is unique in that the maximum Status is also the absence of Pressures. As such, one minus the Status of each pollutant type was used for Pressures data, with the addition of a pressure from shrimp farm pollution (Table S7). Ecological resilience was the same as the global analysis, and the UIE Governance Ranking for Brazilian states was used to assess social resilience (Table S8).

## J. Biodiversity

People attribute value to biodiversity simply for its existence. This goal assesses the conservation status of species based on two sub-goals: Species and Habitats. Species were assessed because they are what one typically thinks of in relation to biodiversity. Because only a small proportion of marine species have been mapped and assessed for their conservation status, we also evaluate Habitats as a proxy for the condition of a broad suite of species that depend on them. We calculate each sub-goal separately and treat them equally when calculating the overall goal score.

**Species sub-goal**

A list of marine species that occur in Brazil and were evaluated globally under the IUCN Red List assessment process was combined with a list of species assessed regionally in Brazil using the same criteria (Brazilian Red List assessments from Chico Mendes Institute for Biodiversity Conservation; see Data Layers). We substitute global assessments for regional (Brazil-specific) assessments whenever these were available. We had assessments for a total of 504 species.

The target for the species sub-goal is to have all species at a risk status of Least Concern. The Status of assessed species was calculated as the threat status-weighted average of all species occurring in the Brazilian EEZ (we did not weight by area of occurrence as in Halpern *et al.* [1] because distribution maps were not available for all species at the time of this assessment). The sub-goal was therefore calculated at the national level, giving equal weight to all species occurring in Brazilian waters. Threat weights were assigned based on the IUCN threat categories status of each *i* species, following the weighting schemes developed by Butchart *et al.* [29] (Table S5). For the purposes of this analysis, we did not include species with the Data Deficient classification following previously published guidelines for a mid-point approach [30]. The Status score in the Species sub-goal (*X_SSP_*) was therefore:

$X_{SPP}=\frac{\sum_{i=1}^{N} w_{i}}{N}$ (Eq. S21)

where *N* is the number of species occurring in Brazil and *w_i_* is the threat weight assigned to each species, where species with high weight are in good condition.

**Table S5. Weights used for threat status weighted-average assessment of Species, based on IUCN threat categories.**

| **Risk Category** | **IUCN code** | **Weight** |
| --- | --- | --- |
| Extinct | EX | 0.0 |
| Critically Endangered | CR | 0.2 |
| Endangered | EN | 0.4 |
| Vulnerable | VU | 0.6 |
| Near Threatened | NT | 0.8 |
| Least Concern | LC | 1.0 |

We calculated Trend as the average of the population trend assessments for all species within Brazil, with species’ trends assigned a value of 0.5 for increasing, 0 for stable, and -0.5 for decreasing using the population trend data associated with the species assessment conducted by IUCN or the Brazilian government. Pressures and Resilience follow Halpern *et al*. [1] with new modeled pressures for Trash, Habitat Destruction (intertidal), and shrimp farming; social pressures and resilience come from the UIE Governance Ranking for Brazilian states (Table S7, Table S8).

**Habitat sub-goal**

The Status of the Habitat sub-goal (X_H_) was assessed for mangroves, coral reefs, seagrass beds, salt marshes, and subtidal soft-bottom habitats following methods outlined elsewhere [1]. Status was assessed as the average of the condition of each *k* habitat (C_k_; measured as loss of habitat and/or percent degradation of remaining habitat), such that:

$X_{H}=\sum_{i=1}^{k} \frac{C_{c}}{C_{r}}$ (Eq. S22)

where C_c_ is the current condition and C_r_ is the reference condition specific to each *k* habitat. The timeframes between current and reference condition vary across habitats, but we generally used a 20 year gap. However, it is important to bear in mind that we were able to obtain only a few time-series in which habitat health was resampled through time, so that information from a few point estimates had to be used to infer the health of a large and highly heterogeneous region (see details discussed above, particularly for coral and seagrasses, and Selig *et al*. [25].

Trend in habitat data were calculated from the slope of the linear trend in extent or condition, depending on habitat type. For coral reefs, we calculated the slope of all sites within Brazil for which multiple-year surveys were conducted (n=11), and then averaged the slopes across sites. For seagrasses, we had monitoring data for only three sites; we therefore chose not to rely on these data to characterize seagrass trends for Brazil, but rather apply the average global trend in seagrass decline (average of 3.5% decline/year from [31]). Data on mangrove extent were available at the state level for 2010, but reference values were available only at the national scale. As the rates of mangrove decline may have significant regional differences within Brazil, we developed a simple linear model correlating national level data on mangrove extent from 1980 – 2010 [21, 22, 32] with increase in coastal urban population density and shrimp farm extent over the same period. Using this model, we predicted the rate of mangrove loss in each coastal state using state level data on current mangrove area [21], urban population density of coastal municipalities and shrimp farm extent. We used these predictions to calculate the recent trend in mangrove for each state. For salt marshes we used trend data for Rio Grande do Sul state [20]. No suitable data were available for Santa Catarina state. We therefore applied the same trend from Rio Grande do Sul. We assume that general trends in salt marsh loss are similar in these two neighboring states. For soft-bottom habitat we use the same data as Halpern *et al.* [1], calculating the slope of the recent change in condition over the past five years, i.e. the change in proportion of catch from trawl fishing per unit area of habitat within Brazil.

Ecological pressures and resilience varied by habitat, but social pressures and resilience were assumed to affect all habitats equally (see Table S7, Table S8). Several resilience measures were included in this goal that were not used in other habitat-based goals. These directly relate to biodiversity conservation, and therefore were included even though they do not explicitly address specific pressures, as do other resilience measures used in this framework (see Table S8).

# 5. Data Layers

This section includes information on new regional data sets that were incorporated into this case-study. Under each data type are listed the original data sources. In addition, summary data tables of the regional datasets used in this case study will be made available at <http://ohi-science.org>. For specific details on global data sets applied to our analysis, refer to Halpern *et al.* [1].

***Coastal land area***

Where used: used with other data layers in a variety of dimensions for several goals.

Description: An ESRI shapefile of the coastal municipalities was obtained from IBGE (*Malha digital dos municípios brasileiros*, 2007; [www.ibge.gov.br](http://www.ibge.gov.br); Accessed February 2010) and used in goal calculations for Clean Waters and Tourism and Recreation. For goals requiring ecological areas, we used fixed-distance coastal buffers based on a high-resolution land-sea model (see Study Area section).

***Coral reefs***

Where used: Status and Trend in Coastal Protection, Carbon Storage and Biodiversity.

Description: Coral reef extent data are derived from the 500m resolution dataset developed for Reefs at Risk Revisited [24]. We calculated extent per state by determining the coral reef area within each state’s coastal waters. Data on percent live coral cover (Reef Check Brazil project [33]) did not meet minimum data requirements (2 or more points between 1980-1995 to calculate a reference condition). We therefore used average values on percent coral cover for adjacent EEZs (countries in the Caribbean). Trend was calculated as an average of the slopes for sites with multiple year monitoring [33]. We used 2006 as the most recent year (post-2006 data were not available at the time of this analysis). Only 11 sites showed multiple year monitoring. The resulting trend was -0.14 over a 5-year period.

***Habitat destruction: intertidal***

Where used: Pressure for several goals

Description: The proxy for intertidal habitat destruction was the population density of coastal municipalities. This is based on the assumption that intertidal habitat destruction is proportional to the density of human populations living along the coast (see [1]). For Brazil, data on urban population in 2010 were obtained for all coastal municipalities from the Brazilian Institute for Geography and Statistics (IBGE). The pressure was assessed either at the level of Brazil EEZ (Fisheries sub-goal of Food Production, and Artisanal Opportunity goal) or at the state level (all other goals). The urban population was summed across the appropriate scale, then divided by the total area of all coastal municipalities, or total area of coastal municipalities within the state, depending on the scale of assessment.

***Hotel jobs***

Where used: Status and Trend for Coastal Tourism and Recreation

Description: The number of people employed in hotel establishments was obtained from the RAIS database (“Relação Anual de Informações Sociais”: www.rais.gov.br). Statistics on employment and commercial establishments are available from 1985 to the most recent year. Trend data were calculated using data from 2007 to 2011. Data are collected and managed by the Brazilian Ministry of Labor and Employment.

***Human population: coastal municipalities***

Where used: Status and Trend for Clean Waters, and proxy for intertidal habitat destruction.

Description: Urban population data were obtained for all coastal municipalities for 1980, 1991, 2000, and 2010 from the Brazilian Institute for Geography and Statistics (IBGE: www.ibge.gov.br). Status calculations used values from 2010 census as the current year, and Trend calculations used estimated values from log-linear models fitted to 1980-2010 data.

***Iconic species list***

Where used: Status and Trend for Iconic Species sub-goal of Sense of Place

Description: The original list of Iconic Species used for Brazil [1] was expanded to include not only WWF recognized priority and flagship species (global designations), but additional species that may be recognized as important to cultural identity, and aesthetic or touristic value. These were selected based on the existence of governmental or non-governmental projects with specific focus on the conservation of these species (Table S6). The IUCN Red List category and population trend for each species was obtained from Brazilian national Red List assessments or global IUCN Red List assessments, when regional evaluations were not available.

**Table S6. Species added to Iconic Species list for Brazil in addition to WWF recognized priority and flagship species, which were included in the global assessment.**

| **Species name** | **Common name** | **Conservation project** |
| --- | --- | --- |
| *Eubalaena australia* | Southern right whale | Projeto Baleia Franca |
| *Megaptera novaeangliae* | Humpback whale | [Instituto Baleia Jubarte](http://www.baleiajubarte.org.br) |
| *Sotalia guianensis* | Guiana dolphin | Instituto Baleia Jubarte |
| *Stenella longirostris* | Spinner dolphin | Projeto Golfinho Rotator |
| *Chelonia mydas* | Green sea turtle | Projeto TAMAR |
| *Lepidochelys olivacea* | Olive ridley sea turtle | Projeto TAMAR |
| *Caretta caretta* | Loggerhead sea turtle | Projeto TAMAR |
| *Eretmochelys imbricata* | Hawksbill sea turtle | Projeto TAMAR |
| *Dermochelys coriacea* | Leatherback turtle | Projeto TAMAR |
| *Epinephelus itajara* | Atlantic goliath grouper | Projeto Meros do Brasil |
| *Pterodroma arminjoniana* | Trindade petrel | Projeto Albatroz |
| *Puffinus lherminieri* | Audubon's shearwater | Projeto Albatroz |
| *Diomedea exulans* | Wandering albatross | Projeto Albatroz |
| *Diomedea dabbenena* | Tristan Albatross | Projeto Albatroz |
| *Diomedea epomophora* | Southern royal albatross | Projeto Albatroz |
| *Diomedea sanfordi* | Northern royal albatross | Projeto Albatroz |
| *Thalassarche melanophrys* | Black-browed albatross | Projeto Albatroz |
| *Thalassarche chlororhynchos* | Atlantic yellow-nosed albatross | Projeto Albatroz |
| *Thalassarche chrysostoma* | Grey-headed albatross | Projeto Albatroz |
| *Phoebetria fusca* | Sooty Albatross | Projeto Albatroz |
| *Macronectes giganteus* | Southern giant petrel | Projeto Albatroz |
| *Fulmarus glacialoides* | Southern fulmar | Projeto Albatroz |
| *Procellaria aequinoctialis* | White-chinned petrel | Projeto Albatroz |
| *Procellaria conspicillata* | Spectacled petrel | Projeto Albatroz |
| *Puffinus gravis* | Great shearwater | Projeto Albatroz |

***Indigenous lands***

Where used: Status for Lasting Special Places sub-goal of Sense of Place

Description: Indigenous lands are designated through the Brazilian federal agency representing indigenous groups, Fundação Nacional do Índio (FUNAI). Shapefiles in ESRI format were obtained through the website of the Brazilian system of protected areas (CNUC; see Marine and Terrestrial Protected Areas). Indigenous lands receive a separate designation, as they are set aside for the exclusive use of indigenous peoples.

***Mangroves***

Where used: Status and Trend in Coastal Protection, Carbon Storage and Biodiversity.

Description: Data on the current extent of mangroves (year 2010) were obtained at the state level from a report compiled by the Ministry of the Environment [21]. Data on historic mangrove extent were available only at the national level [22,32], spanning years 1980 to 2000. As the rates of mangrove decline may have significant regional differences within Brazil, we developed a simple model correlating national level data on mangrove extent from 1980 – 2010 [21, 22, 32] with increase in coastal urban population density and shrimp farm extent over the same period. Using the model parameters obtained at national level, we then predicted the rate of mangrove loss in each coastal state using state level data on current mangrove area [21], urban population density of coastal municipalities and shrimp farm extent.

***Mariculture yield***

Where used: Status and Trend for Mariculture sub-goal of Food Provision

Description: Mariculture harvests in tons were obtained from fisheries statistics reports from the Brazilian Institute of the Environment and Renewable Natural Resources (IBAMA) for years 2001-2007 ([www.ibama.gov.br/documentos-recursos-pesqueiros/estatistica-pesqueira](http://www.ibama.gov.br/documentos-recursos-pesqueiros/estatistica-pesqueira)). These reports provide a breakdown of landings by species by Brazilian state. More recent reports from the Brazilian Ministry of Fisheries (2008 onwards) aggregate data across all of Brazil when reporting for species and across all species when reporting at state level, and could not be used. Low landings of finfish were reported only for Sergipe State (SE) in 2002 and 2003, and were removed from the analysis. Similarly, scallop landings appeared sporadically in only a few states across the time series, and were also not included in the model.

***Marine and terrestrial protected areas (coastal and EEZ)***

Where used: Status for Lasting Special Places sub-goal of Sense of Place, Resilience measures

Description: These data are from the Brazilian Ministry of the Environment’s database on the national system of protected areas (Cadastro Nacional de Unidades de Conservação (CNUC): www.mma.gov.br/areas-protegidas/cadastro-nacional-de-ucs/dados-georreferenciados). The data are available as ESRI shapefiles and include the names, designation year between 1914-2010, and protected area category of municipal, state and federal protected areas. There are 12 categories of protection within CNUC, which fall under two broad groups: fully protected, and sustainable use. The category “Área de Proteção Ambiental (APA)” was removed from the analysis, as these represent large areas used for multiple-use area zoning and do not capture the sense of “special place” intended with this goal.

***Marine species***

Where used: Status and Trend for Species sub-goal of Biodiversity; ecological integrity Resilience measure for several goals.

Description: A list of marine species that occur in Brazil and were evaluated globally under the IUCN Red List assessment process was combined with a list of species assessed regionally in Brazil using the same criteria (Brazilian Red List assessments from Chico Mendes Institute for Biodiversity Conservation, ICMBio). For all species occurring in both lists (n=100), we used regional (Brazil-specific) status categories. A total of 200 new species of marine fishes (Actinopterygii) from regional assessments were added, for a combined total of 504 species. At the time of this analysis, regional assessments for all species, except marine turtles had not yet undergone the final consistency checking process mandated by IUCN, thus final categories within Brazil are subject to change. Species listed as Data Deficient were considered as Not Evaluated for this analysis.

***Pathogen pollution***

Where used: Status, Trend and Pressure for Clean Waters, Pressure for several other goals

Description: Data on the presence or absence of sewage treatment services in Brazilian coastal municipalities in 2008 (Brazilian Institute of Geography and Statistics, IBGE, [www.ibge.gov.br/home/estatistica/populacao/condicaodevida/pnsb2008/default.shtm](http://www.ibge.gov.br/home/estatistica/populacao/condicaodevida/pnsb2008/default.shtm)), was used in conjunction with urban population data from coastal municipalities to determine the percent coastal population per state without access to improved sanitation. This was combined with information on population density to create a proxy for pathogens in coastal waters (see [1]). As data on sewage treatment were available from IBGE only for 2008, we used a time-series on access to sanitation in Brazil to calculate Trend (data from WHO/UNICEF Joint Monitoring Programme, [www.wssinfo.org](http://www.wssinfo.org)). Using this, we estimate the recent rate of change in sewage access and applied this as a discount factor to the estimated coastal population density in 2005 and 2010, based on a linear model using urban population data in 1991, 2000, 2010 (IBGE).

***Salt marsh***

Where used: Status and Trend in Coastal Protection, Carbon Storage and Biodiversity.

Description: Data on current salt marsh extent (2010 values) were obtained at the state level from a report compiled by the Ministry of the Environment [21]. Data were listed under the category “marismas” (salt marshes) and indicate this habitat occurs in Rio Grande do Sul and Santa Catarina states only. Trend data for Rio Grande do Sul were obtained from [20] and show a stable trend (i.e. no change in extent) over the past 25 years. We applied the same trend to the neighboring state of Santa Catarina, as no other information was available.

***Seagrass***

Where used: Status and Trend in Coastal Protection, Carbon Storage and Biodiversity.

Description: Seagrass extent was calculated from vector-based data from the Global Distribution of Seagrasses [23]. The total extent for Brazil was allocated on a per-state basis based on coastline length because higher resolution data, or models to predict seagrass distribution were not available. Due to limited monitoring sites for seagrasses within Brazil (only three sites monitored using the same protocols, [34]), we chose to use global values for seagrass trends from [31].

***Shrimp farm extent***

Where used: Pressure for Clean Waters goal

Description: Data on the extent of coastal shrimp farms (km^2^) in 2010 for each state was obtained from the Ministry of the Environment [21] and divided by coastal area (1 km inland buffer) of each state to estimate the polluting pressure of this activity.

***Shrimp farm expansion***

Where used: Pressure for several goals

Description: The percent increase in extent of coastal shrimp farms (km^2^) for each state was calculated from 2000 to 2010 using data from the Brazilian Association of Shrimp Farmers for 2000 reported in Oliveira *et al*. [35] and from the Ministry of the Environment for 2010 [21].

***Trash pollution***

Where used: Status and Pressure for Clean Waters, Pressure for several other goals

Description: Data on the presence or absence of four types of solid waste management were obtained from the Brazilian Institute of Geography and Statistics (IBGE, <http://www.ibge.gov.br/home/estatistica/populacao/condicaodevida/pnsb2008/default.shtm>), namely: beach clean-up services, household garbage collection, household recycling collection, and garbage collection in city streets. These data were used to calculate the percent urban population without access to each type of service, which was combined with information on population density to create a proxy for trash (marine debris) input. For Trend, we used the global assessment for Brazil which was < -0.01 [1].

***UIE Ranking of Management for Brazilian States***

Where used: Social resilience and Pressure measure for all goals.

Description: We used a metric developed by The Economist Intelligence Unit (UIE), which ranks management effectiveness in Brazilian states for 2011 (<http://veja.abril.com.br/multimidia/infograficos/ranking-de-gestao-dos-estados-brasileiros-2011>). The index is comprised of eight categories: Political Environment, Economic Environment, Tributary and Regulatory Environment, Policies for International Investment, Human Resources, Infrastructure, Innovation, and Sustainability. Values for each category and for the aggregate score are reported by UIE on a scale of 0 to 100. We used the aggregate score for each coastal state. A complete list of indicators and methods for calculating the UIE index are available at: <http://veja.abril.com.br/infograficos/clp/levantamentos-e-metodologia.pdf>.

# 6. Supporting Figures and Tables

**Figure S1. Major country regions of Brazil with areas considered to be Special Places (protected areas and Indigenous lands). The reference value for this sub-goal was 30% protection within the coastal zone (1 km inland and 3 nmi offshore; delineated by nearshore black line). The category “Área de Proteção Ambiental” was excluded from our analysis.**


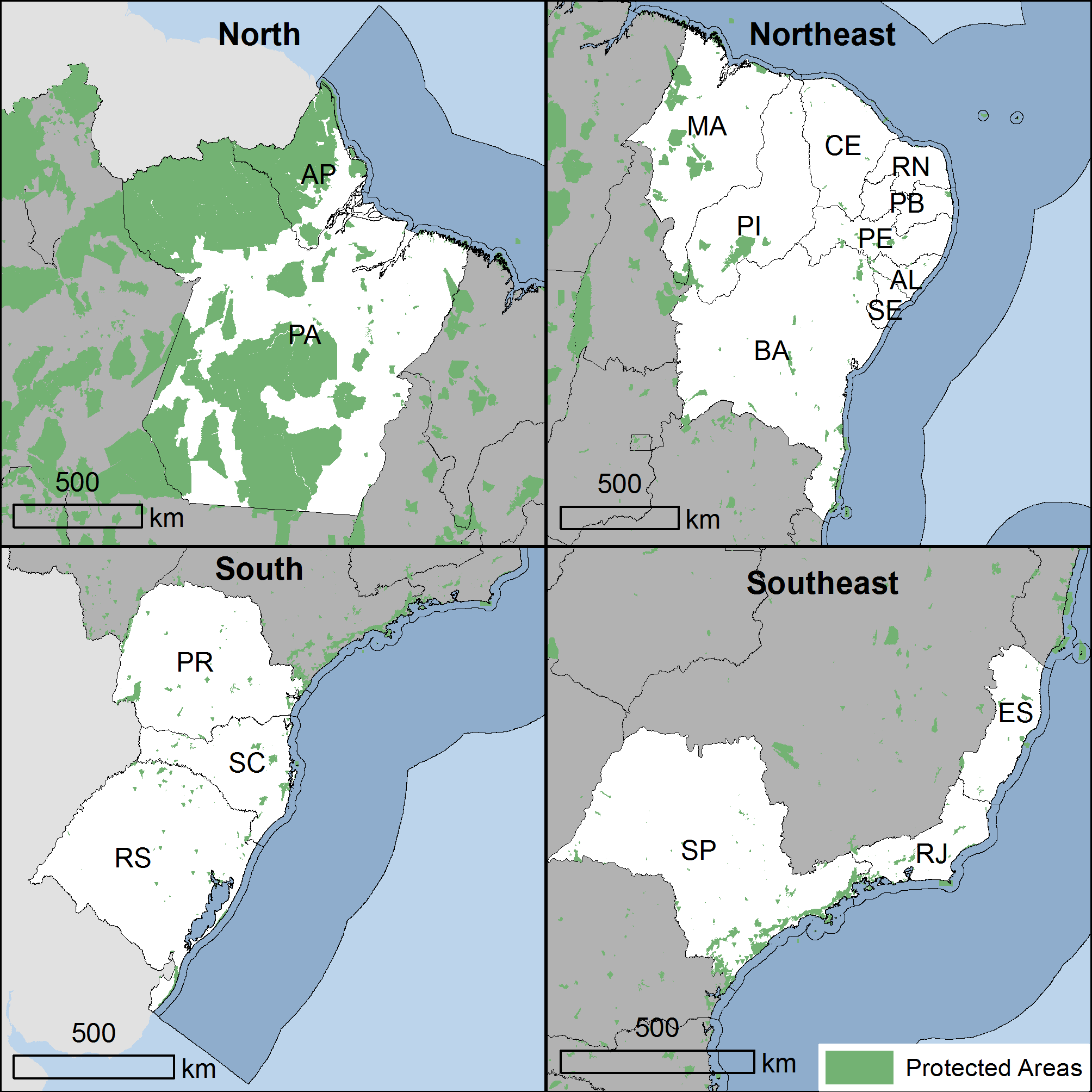
**Figure S2. Total biomass of wild capture fisheries landings and mMSY for the main portion of the Brazilian EEZ (Brazil coast), and for the EEZ area surrounding Trindade and Martim Vaz islands, a separate reporting unit. The dotted lines represent a 70-80% buffer around mMSY.**

**Table S7. Matrix of pressure rankings for all goals. The rankings are used to determine the relative contribution of the pressure scores to the overall ecological pressure score (p_E_). Social pressures (p_S_) were calculated using a single index (UIE) and thus require no weighting.**

**Table S7. (continued)**

**Table S8. Matrix of data used for the Resilience measure for each of the goals. The versions of fishing resilience use different combinations of metrics relating to habitat protection, percent marine protected area coverage, and fisheries management effectiveness (refer to Halpern *et al.* 2012[1]).**

**
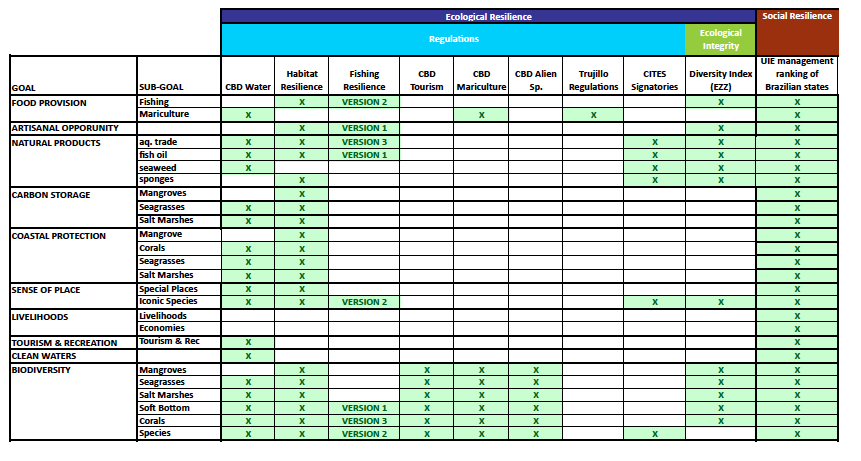
**

# 6. References

1. Halpern BS, Longo C, Hardy D, McLeod KL, Samhouri JF, et al. (2012) An index to assess the health and benefits of the global ocean. Nature 488: 615-622.

2. Samhouri JF, Lester SE, Selig ER, Halpern BS, Fogarty MJ, et al. (2012) Sea sick? Setting targets to assess ocean health and ecosystem services. Ecosphere 3(5):41.

3. Perrings C, Naeem S, Ahrestani F, Bunker DE, Burkill P, et al. (2010) Ecosystem services for 2020. Science 330:323.

4. Perrings C, Naeem S, Ahrestani F, Bunker DE, Burkill P, et al. (2011) Ecosystem services, targets, and indicators for the conservation and sustainable use of biodiversity. Frontiers in Ecology and the Environment 9:512-520.

5. CBD (2010) Strategic plan for biodiversity 2011-2020, including Aichi biodiversity targets. Available from: <http://www.cbd.int/sp/targets>. Accessed 5 November 2012.

6. Freire KMF, Oliveira TLS (2007) Reconstructing catches of marine commercial fisheries for Brazil, p. 61-68 In: Zeller D, Pauly D, editors. Reconstruction of marine fisheries catches for key countries and regions (1950-2005) Fisheries Centre Research Reports 15(2). Fisheries Centre, University of British Columbia, Vancouver.

7. Kleisner K, Pauly D (2011) Stock-catch status plots of fisheries for Regional Seas. In: The state of biodiversity and fisheries in Regional Seas. Fisheries Centre Research Reports 19:37-40. Fisheries Centre, University of British Columbia, Vancouver.

8. Branch TA, Hively DJ, Hilborn R (2013) Is the ocean food provision index biased? Nature 495: E5-E6.

9. Halpern BS, Gaines SD, Kleisner K, Longo C, Pauly D, et al. (2013) Halpern et al. reply. Nature 495: E7.

10. FAO (2010) The state of world fisheries and aquaculture 2010. Food and Agriculture Organization of the United Nations. Rome. 197 p.

11. Srinivasan UT, Cheung WWL, Watson R, Sumaila UR (2010) Food security implications of global marine catch losses due to overfishing. Journal of Bioeconomics 12: 183–200. DOI:10.1007/s10818-010-9090-9

12. Costello C, Ovando D, Hilborn R, Gaines SD, Deschenes O, et al. (2012) Status and solutions for the world’s unassessed fisheries. Science 338:517-520.

13. Martell S, Froese R (2012) A simple method for estimating MSY from catch and resilience. Fish and Fisheries. DOI: 10.1111/j.1467-2979.2012.00485.x

14. Costello C, Deschênes O, Larsen A, Gaines S (2013) Removing biases in forecasts of fishery status. Journal of Bioeconomics*.* DOI 10.1007/s10818-013-9158-4

15. Srinivasa UT, Cheung WWL, Watson RA, Sumaila UR (2013) Response to removing biases in forecasts of fishery status. Journal of Bioeconomics. DOI 10.1007/s10818-013-9160-x

16. Trujillo P (2008) The performance of 53 countries in managing marine resource Alder J, Pauly D, editors. Fisheries Centre Research Reports, University of British Columbia, Vancouver, Canada.

17. Guimarães AS (2005) Carcinicultura marinha brasileira: sustentabilidade, reflexões históricas e situação atual. Monograph. Universidade Federal de Pernambuco, Departamento de Oceanografia, Especialização em Gestão de Ambientes Costeiros Tropicais. 86 pp.

18. MPA (2011) Registro geral de pescadores ganha maior transparência e pode ser acessado pela internet. Ministério da Pesca e Aquicultura. Available at: <http://www.brasil.gov.br/noticias/arquivos/2011/04/27/governo-disponibiliza-acesso-ao-registro-geral-de-pescadores-e-cancela-mais-de-70-mil-carteiras>. Accessed 5 December 2012.

19. Cordell J (2006) Brazil: Dynamics and challenges of marine protected areas development and coastal protection. In: Scaling up marine management: The role of marine protected areas. World Bank, Washington DC, pp 58-77.

20. Marangoni JC, Costa CSB (2009) Natural and anthropogenic effects on salt marsh over five decades in the Patos Lagoon (Southern Brazil). Brazilian Journal of Oceanography 57: 345-350.

21. MMA (2010) Panorama da conservação dos ecossistemas costeiros e marinhos no Brasil. Gerência de Biodiversidade Aquática e Recursos Pesqueiros. Ministério do Meio Ambiente. Brasília. 148 p.

22. FAO (2007) The world’s mangroves 1980-2005. FAO Forestry Paper 153. Food and Agriculture Organization of the United Nations, Rome.

23. UNEP-WCMC (2005) Global Distribution of Seagrasses-Points Dataset. Available at: <http://data.unep-wcmc.org/datasets/9>. Accessed 5 December 2012.

24. Burke L, Reytar K, Spalding M, Perry A (2011) Reefs at Risk Revisited. World Resources Institute, Washington D.C. Available at: <http://www.wri.org/publication/reefs-at-risk-revisited>. Accessed 5 December 2012.

25. Selig ER, Longo CS, Halpern BS, Best BD, Hardy D, et al. (2013) Assessing global marine biodiversity status within a coupled socio-ecological perspective. PLOS One 8(4): e60284

26. Duke NC (1996) Mangrove reforestation in Panama: an evaluation of planting in areas deforested by a large oil spill. In: Field C, editor. Restoration of mangrove ecosystems. International Society for Mangrove Ecosystems ISME and International Tropical Timber Organisation ITTO. Okinawa, Japan, pp. 209-232.

27. Oliveira AVM, Vassallo M (2007) Estudos da competitividade do turismo brasileiro. Determinantes da demanda dos turistas que viajam pelo Brasil. Ministério do Turismo, Brasília, Brazil. 39 pp.

28. Halpern BS, Walbridge S, Selkoe KA, Kappel CV, Micheli F, et al. (2008) A global map of human impact on marine ecosystems. Science 319: 948-952.

29. Butchart SHM, Resit Akçakaya H, Chanson J, Baillie JEM, Collen B, et al*.* (2007) Improvements to the Red List Index. PLoS ONE 2: e140.

30. IUCN (2011) Guidelines for appropriate uses of the IUCN Red List Data. Incorporating the guidelines for reporting on threatened and the guidelines on scientific collecting of threatened species. Version 2. Adopted by the IUCN Red List Committee and IUCN SSC Steering Committee. Available from: <http://intranet.iucn.org/webfiles/doc/SpeciesProg/RL_Guidelines_Data_Use.pdf>. Accessed 15 June 2012.

31. Duarte CM, Dennison WC, Orth RJW, Carruthers TJB (2008) The charisma of coastal ecosystems: addressing the imbalance. Estuaries and Coasts 31: 233-238.

32. Giri C, Ochieng E, Tieszen LL, Zhu Z, Singh A, et al*.* (2011) Status and distribution of mangrove forests of the world using earth observation data. Global Ecology and Biogeography 20:154-159.

33. MMA (2006) Monitoramento dos recifes de coral do Brasil. Ferreira BP, Maida M, editors. Ministério do Meio Ambiente. Brasília. 250 p

34. Short FT, Polidoro B, Livingston SR, Carpenter KE, Bandeira S, et al. (2011) Extinction risk assessment of the world’s seagrass species. Biological Conservation 144: 1961-1971.

35. Oliveira VG, Jerônimo CEM, Cezar GM, Santiago AF Jr., de Sousa Melo HN, et al. (2002) Proposta para minimização do impacto causado pela carcinicultura nos manguezais do RN. Abstract. 28th Interamerican Congress of Sanitary and Environmental Engineering, 27-31 October 2002, Cancún, Mexico.
